# A Systematic Study of Liquid Chromatography in Search of the Best Separation of Cannabinoids for Potency Testing of Hemp-Based Products

**DOI:** 10.3390/molecules30040758

**Published:** 2025-02-07

**Authors:** Ayowole Owolabi, Olalekan Ogunsola, Emma Joens, Medline Kotler, Liguo Song

**Affiliations:** Department of Chemistry, Western Illinois University, Macomb, IL 61455, USA; aa-owolabi@wiu.edu (A.O.); om-ogunsola@wiu.edu (O.O.); ep-joens@wiu.edu (E.J.); mc-kotler@wiu.edu (M.K.)

**Keywords:** potency testing, cannabinoids, hemp, stationary phase, chromatographic separation

## Abstract

A study was conducted to search for the best separation of eighteen cannabinoids, the maximum number of cannabinoids that have been quantified so far, for potency testing of hemp-based products using liquid chromatography diode array detector (LC-DAD). The investigation utilized four column types, all sharing the same dimension (150 mm × 2.1 mm) and core–shell particle size (2.7 µm), but different stationary phases: dimethyl-octadecyl (Poroshell 120 EC-C18), diisobutyl-octadecyl (Raptor ARC-18), reverse phase (RP)-carbamate (Cortecs Shield RP-18), and RP-amide (Ascentis Express RP-Amide). The resolution of adjacent cannabinoids was kept close to 1.5 or higher, while the separation time was kept as short as possible. The fastest separation was achieved within 15.0 min using two sequential Raptor ARC-18 columns, with a mobile phase consisting of 75.0% acetonitrile and 25.0% aqueous solution of 0.03% formic acid and 0.5 mM ammonium formate at pH 2.97, at a flow rate of 0.5 mL/min. A slightly improved resolution of the eighteen cannabinoids was obtained within 18.5 min using two sequential Poroshell 120 EC-C18 columns under similar conditions, except for a mobile phase containing 77.5% acetonitrile and a reduced flow rate of 0.45 mL/min due to backpressure higher than 600 bars. Furthermore, a rapid 7.0 min separation was achieved for potency testing of hemp-based products by liquid chromatography electrospray ionization tandem mass spectrometry (LC-ESI/MS/MS) using a Cortecs Shield RP-18 column, with a mobile phase consisting of 70.0% acetonitrile and 30.0% aqueous solution of 0.01% formic acid and 1 mM ammonium formate at pH 3.38 at a flow rate of 0.5 mL/min.

## 1. Introduction

Cannabinoids are a structurally diverse class of organic molecules that are uniquely produced by the plant *Cannabis sativa* L. [1]. So far, more than 150 cannabinoids have been isolated and identified [2,3]. They can be classified into three main groups: acidic ones that are enzymatically biosynthesized by the plant; neutral ones that are directly decarboxylated from the acidic ones by heat, either in the plant or after harvest; and others, including both acidic and neutral ones, that are formed from other non-enzymatic processes, e.g., isomerization, decomposition, and/or degradation [2,3,4].

The plant *Cannabis sativa* L. has two primary varieties [1]. *Cannabis sativa* L. var. *indica* is generally known as marijuana, which produces high content of Δ^9^-tetrahydrocannabinol (THC), the infamous psychotropic cannabinoid. *Cannabis sativa* L. var. *sativa* is commonly referred to as hemp, which produces high content of cannabidiol (CBD), the primary anti/non-psychotropic cannabinoid. In the USA, the Controlled Substances Act (CSA) of 1970 classified all parts of the plant *Cannabis sativa* L., including both marijuana and hemp, as a Schedule I controlled substance. However, the 2018 Farm Bill legalized hemp as products with a Δ^9^-THC concentration of not more than 0.3% (*w*/*w*) on a dry weight basis. Since then, a variety of hemp-based products, including plant materials, concentrates, vape cartridges, tinctures, edibles, topicals, and pet treats, entered the U.S. market, presumably due to the health benefits of cannabinoids in hemp, predominantly CBD [5,6,7].

Potency testing of hemp-based products aims to quantify all of the major cannabinoids in a sample so that their quality and safety can be determined. Since 2017, a working group of the Stakeholder Panel on Strategic Food Analytical Methods of the AOAC International have developed Standard Method Performance Requirements (SMPRs) for potency testing of cannabinoids in cannabis concentrate [8], dried plant materials [9], plant materials of hemp [10], edible chocolates [11], and beverages [12]. Five cannabinoids (i.e., CBD, cannabidiolic acid (CBDA), cannabinol (CBN), Δ^9^-THC, and Δ^9^-tetrahydrocannabinolic acid (THCA)) are in the required list, while nine cannabinoids (i.e., cannabigerol (CBG), cannabigerolic acid (CBGA), cannabichromene (CBC), cannabichromenic acid (CBCA), cannabidivarin (CBDV), cannabidivarinic acid (CBDVA), Δ^8^-THC, tetrahydrocannabivarin (THCV), and tetrahydrocannabivarinic acid (THCVA)) are in the desirable list, to be quantified. Quantification of other cannabinoids is not required, probably based on an assumption that their contents are lower than the required limit of quantification (LOQ).

Both gas chromatography (GC) and liquid chromatography (LC) have been extensively utilized for analyzing bioactive compounds [13,14], including cannabinoids [15,16,17,18]. Due to the coexistence of both acidic and neutral cannabinoids in hemp-based products, it has been well acknowledged that their potency testing preferred LC to GC because of the decarboxylation of acidic cannabinoids under GC conditions. Regarding LC detection, a diode array detector (DAD) was preferred to electrospray ionization tandem mass spectrometry (ESI/MS/MS) due to its wide accessibility by crime labs, commercial suppliers, and farmers. However, the selectivity of LC-DAD relies on achieving baseline separation of all cannabinoids, including unknown compounds exceeding the required LOQ in a sample. Therefore, the LC-DAD method development must encompass all cannabinoids previously quantified in hemp-based products. To date, the maximum number of cannabinoids quantified for potency testing in hemp-based products is eighteen [19], which included the fourteen cannabinoids listed by the AOAC international, cannabinolic acid (CBNA) and cannabicitran (CBT) that were often found in the samples, and cannabicyclolic acid (CBLA) and cannabicyclol (CBL) that were occasionally found in the samples (Supplementary Figure S1). In addition, LC-DAD methods preferred isocratic to gradient separation because mobile phase change would result in baseline drift, due to detection of most neutral cannabinoids at 230 nm or shorter wavelengths, and subsequently higher LOQs. Although it is well known that acetonitrile has a UV cutoff of 190 nm, a baseline drift during a gradient separation at 230 nm has been recently reported and proven problematic [20]. In comparison with LC-DAD, LC-ESI/MS/MS only requires baseline separation of structural isomers of the eighteen cannabinoids, therefore promising shorter analytical time [19,20,21]. Additionally, gradient separation would not cause baseline drift and subsequently higher LOQs [19,20,21]. Therefore, separations achieved using LC-DAD methods can be readily adapted for LC-ESI/MS/MS methods, but the reverse is not applicable.

So far, many methods have been published for potency testing of hemp-based products [15,16]. However, only five LC-DAD methods (Supplementary Table S1) were validated for fourteen and more cannabinoids [19,20,22,23,24]. Among them, three used isocratic separation [19,22,23], and only two achieved baseline separation of all cannabinoids [19,23]. Although four LC-ESI/MS/MS (Supplementary Table S2) [21,25,26,27,28] and two LC-ESI/MS methods (Supplementary Table S3) [29,30] were also validated for fourteen and more cannabinoids, none of them achieved baseline separation of all cannabinoids, and only two of them used isocratic elution [21,29]. Notably, one LC-ESI/MS/MS method failed to achieve baseline separation of Δ^9^-THC and Δ^8^-THC [28], but a recent study using a quadrupole time-of-flight (QTOF) mass spectrometer for targeted analysis demonstrated that multiple reaction monitoring (MRM) could not definitively distinguish between Δ^9^-THC and Δ^8^-THC [21].

The objective of this study was to search for the best separation of cannabinoids for potency testing of hemp-based products. Eighteen cannabinoids (Supplementary Figure S1), ten neutral and eight acidic, which were the same as previously listed, were used to discover the best separation.

## 2. Results and Discussion

Four types of columns, with the same inner diameter (2.1 mm) but varied stationary phases, were selected. Two recent reviews by Nahar et al. [15,16] detailed columns used by published methods for the quantification of cannabinoids. Most published methods used columns based on traditional dimethyl-octadecyl (-OSi(CH_3_)_2_C_18_H_37_) stationary phase. Popular choices included Poroshell 120 EC-C18, Ascentis Express C18, and Kinetex C18. Both Poroshell 120 EC-C18 and Ascentis Express C18 used the popular 2.7 µm core–shell particles, while Kinetex C18 used 2.6 µm core–shell particles. Poroshell 120 EC-C18 was selected because its characteristics were publicly available in more detail. Raptor ARC-18, which also used the popular 2.7 µm core–shell particles, was then selected, because it was as popular as Poroshell 120 EC-C18. In addition, its stationary phase was sterically protected C18, i.e., diisobutyl-octadecyl (-OSi(*i*Bu)_2_C_18_H_37_), so it became important to examine their differences in selectivity of cannabinoids. Columns based on polar-embedded reverse-phase (RP) stationary phases were used less frequently, so their selectivity of cannabinoids became highly desirable to be examined in detail. Therefore, Cortecs Shield RP-18 and Ascentis Express RP-Amide were selected. Both used the popular 2.7 µm core–shell particles, but their stationary phases differed in embedded polar functional groups, i.e., RP-carbamate (-OSi(CH_3_)_2_C_3_H_6_O(CO)NHC_12_H_25_) versus RP-amide (-OSi(CH_3_)_2_C_3_H_6_NH(CO)C_15_H_31_). It is noted that the embedded polar functional group of Cortecs Shield RP-18 is not officially documented, but the embedded polar functional group of Symmetry Shield RP-18 is well known as carbamate.

In our preliminary studies, the water/methanol eluting system did not show any resolution of the Δ^8^-THC/CBL pair on the Raptor ARC-18 and Cortecs Shield RP-18 stationary phases, even though low resolution of the Δ^8^-THC/CBL pair was achieved on the Poroshell 120 EC-C18 and Ascentis Express C18 stationary phases. Therefore, in this study, the separation optimization was based on the water/acetonitrile eluting system using isocratic elution. First, baseline separation of the ten neutral cannabinoids using each stationary phase was obtained after the optimum acetonitrile content was determined, which led to good separation of the eight acidic cannabinoids in neutralized forms. Then, good separations of the eighteen cannabinoids altogether using each stationary phase was achieved by adjusting the aqueous solvent pH to change the retention times of the acidic cannabinoids so that coelutions of acidic and neutral cannabinoids were avoided.

### 2.1. Separation of the Ten Neutral Cannabinoids by LC-DAD

Separation optimization started by using 0.02% (*v*/*v*) HCO_2_H as the A solvent, acetonitrile as the B solvent, and 75.0% (*v*/*v*) B in the mobile phase at 0.3 mL/min. Additional %B, either higher than 75.0% (i.e., 77.5 and 80.0%) or lower than 75.0% (i.e., 70.0, 65.0, 60.0, and 57.5%) was then experimented on one by one, if the previous one predicted an acceptable run time of the next one, i.e., neither too short nor too long.

LC-UV separation of the ten neutral cannabinoids was initially performed using a Poroshell 120 EC-C18 column and 80.0, 77.5, 75.0, 70.0, and 65.0% (*v*/*v*) acetonitrile in the mobile phase. As expected, lower acetonitrile content resulted in longer run time. Baseline separation of the ten neutral cannabinoids, i.e., resolution of the adjacent pair is equal or greater than 1.5 (R ≥ 1.5), was achieved using both 75.0 and 70.0% (*v*/*v*) acetonitrile in the mobile phase. Next, LC-UV separation of the ten neutral cannabinoids was carried out using a Raptor ARC-18 column and 77.5, 75.0, 70.0, 65.0, and 60.0% (*v*/*v*) acetonitrile in the mobile phase. The best separation was achieved using 70.0% (*v*/*v*) acetonitrile in the mobile phase, but baseline separation of the CBD/CBG pair was not achieved (R = 1.24). Then, LC-UV separation of the ten neutral cannabinoids was evaluated using a Cortecs Shield RP-18 column and 77.5, 75.0, 70.0, 65.0, 60.0, and 57.5% (*v*/*v*) acetonitrile in the mobile phase. Baseline separation of the ten neutral cannabinoids was achieved using both 65.0 and 57.5% (*v*/*v*) acetonitrile in the mobile phase. However, CBC eluted earlier than CBT using 65.0% (*v*/*v*), but their elution order was reversed using 57.5% (*v*/*v*) acetonitrile in the mobile phase. Finally, LC-UV separation was assessed using an Ascentis Express RP-Amide column and 80.0, 77.5, 75.0, 70.0, and 65.0% (*v*/*v*) acetonitrile in the mobile phase. Baseline separation of the ten neutral cannabinoids was achieved using 65.0% (*v*/*v*) acetonitrile in the mobile phase.

**Figure 1:**
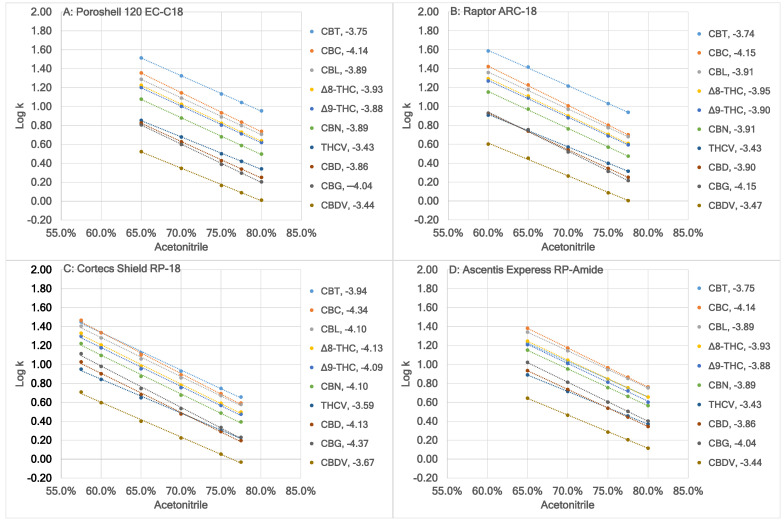
A comparison of Log k of the ten neutral cannabinoids at different acetonitrile content in the mobile phase using four different columns. The A solvent was 0.02% (*v*/*v*) HCO_2_H. The B solvent was acetonitrile. Each cannabinoid is represented by a distinct color, displayed on the right side of each sub-figure along with the slope of the linear regression between Log k and acetonitrile content. In sub-figure (**A**), the linear regression curves follow the same rising sequence as the cannabinoids. However, in sub-figures (**B**–**D**), variations occur due to the crossover of the linear regression curves for CBG/CBD/THCV and CBC/CBT. The lowest correlation coefficient of these plots was 0.9980.

Figure 1 shows a comparison of Log k of the ten neutral cannabinoids, where k was the retention factor, calculated by using (t_r_ − t_m_)/t_m_, with t_r_ and t_m_ being the retention time of individual cannabinoid and the dead time, respectively, at different acetonitrile content using the four columns. It was noted that the retention factor of eight cannabinoids, excluding CBG and CBT (more about them later), increased in the flowing order: Cortecs Shield RP-18, Raptor ARC-18, Poroshell 120 EC-C18, and Ascentis Express RP-Amide, which is exemplified in Table 1 when the mobile phase contained 70.0% (*v*/*v*) acetonitrile. It was believed that this generally indicated the hydrophobic order of the columns. Supplementary Table S4 shows a comparison of the theoretical plate numbers (N) for the ten neutral cannabinoids across four columns when the mobile phase contained 70.0% (*v*/*v*) acetonitrile, calculated using the formula 5.54t_r_^2^/w_1/2_^2^, where w_1/2_ represents the full width at half maximum (FWHM). The results suggest that the four columns had comparable separation efficiency.

As predicted by chromatographic theory, Log k and acetonitrile content had inverse relationships for all cannabinoids using all four columns (Figure 1). Changes in selectivity (also known as separation factor or relative retention, calculated by using (t_r2_ − t_m_)/(t_r1_ − t_m_), with t_r2_ and t_r1_ being the retention time of later and earlier eluting peak, respectively) among adjacent cannabinoids were observed due to the changes in the corresponding slopes, which are listed in the graphs behind cannabinoids. Especially, the elution order of adjacent cannabinoids reversed when two adjacent linear curves crossed each other.

The two traditional RP stational phases, i.e., dimethyl-octadecyl and diisobutyl-octadecyl, resembled each other closely: (1) the elution orders of the ten neutral cannabinoids were the same, and (2) the slopes of the ten neutral cannabinoids were close to each other (Figure 1A,B). However, Poroshell 120 EC-C18 showed higher selectivity of the two critical pairs, i.e., CBG/CBD and Δ^9^-/Δ^8^-THC, to be separated. Using values in Table 1, the selectivity of CBG/CBD and Δ^9^-/Δ^8^-THC was calculated to be 1.069 and 1.051, respectively, for Poroshell 120 EC-C18, but 1.051 and 1.044, respectively, for Raptor ARC-18. The significantly lower selectivity of the CBG/CBD pair by Raptor ARC-18 was responsible for its worse than baseline resolution with all acetonitrile contents in the mobile phase. Poroshell 120 EC-C18 also showed higher selectivity of the CBL/CBC pair, although their resolution did not pose a problem for Raptor ARC-18.

For the two polar-embedded RP stationary phases, i.e., RP-carbamate and RP-amide, the elution orders of the neutral cannabinoids were the same, excluding CBT (more about it later). The slopes of the neutral cannabinoids were higher for Cortecs Shield RP-18 than Ascentis Express RP-Amide, but the differences were approximately the same for each cannabinoid, excluding CBG, CBD, and THCV (more about them next).

Among the ten neutral cannabinoids, the CBG/CBD/THCV pairs were identified as the critical pairs to be separated by all four columns. For the two traditional RP stationary phases (Figure 1A,B), as the acetonitrile content decreased, the selectivity of the CBG/CBD/THCV pairs decreased, and the elution order (i.e., CBG, CBD, and THCV) could be reversed due to the crossover of curves if lower acetonitrile contents were not prohibitive to be experimented due to extremely long run times. This trend appeared to continue by the two polar-embedded RP stationary phases (Figure 1C,D), but at higher acetonitrile contents, until the total reversal of the elution order (i.e., THCV, CBD, and CBG). The acetonitrile content where the CBG/THCV curves crossed over were approximately less than 60.0, 65.0, 77.5, and higher than 80.0% (*v*/*v*) for Poroshell 120 EC-C18, Raptor ARC-18, Cortecs Shield RP-18, and Ascentis Express RP-Amide, respectively.

The selectivity of the Δ^9^-/Δ^8^-THC pair was also obviously different for the two traditional RP and the two polar-embedded RP stationary phases. Using the values in Table 1, it was calculated to be 1.044, 1.051, 1.059, and 1.081 for Raptor ARC-18, Poroshell 120 EC-C18, Cortecs Shield RP-18, and Ascentis Express RP-Amide, respectively.

It was also obvious that the two traditional RP and the two polar-embedded RP stationary phases had different selectivity of the CBC/CBT pair. For the two traditional RP stationary phases (Figure 1A,B), CBT had the highest retention factor of the ten neutral cannabinoids, which were also significantly higher than the retention factor of CBC, the second highest retention factor of the ten neutral cannabinoids. This trend was significantly weakened by the RP-carbamate stationary phase (Figure 1C): the retention factors of CBT were slightly higher than these of CBC with 77.5, 75.0, 70.0, and 65.0% (*v*/*v*), the same as that of CBC with 60% (*v*/*v*), but slightly lower than that of CBC with 57.5% (*v*/*v*) acetonitrile in the mobile phase. The trend was further significantly weakened by the RP-amide stationary phase (Figure 1D): the retention factor of CBT was the same as that of Δ^8^-THC with 80.0, 77.5 and 75.0%, but slightly lower than those of Δ^8^-THC with 70.0 and 65.0% (*v*/*v*) acetonitrile in the mobile phase, which basically annulled the significantly better selectivity of the Δ^9^-/Δ^8^-THC pair offered by the RP-amide over other stationary phases.

### 2.2. Separation of the Eight Acidic Cannabinoids by LC-DAD

Once a good separation of their neutral counterparts was achieved, a good separation of the neutralized forms of the eight acidic cannabinoids, in acidified mobile phases, would be likely. To neutralize the eight acidic cannabinoids in the mobile phases, further separation optimization still used 0.02% (*v*/*v*) HCO_2_H as the A solvent and acetonitrile as the B solvent. A mixture solution containing ten neutral cannabinoids and eight acidic cannabinoids at 1 µg/mL was separated. The effect of acetonitrile content on the separation of the eight acidic cannabinoids using the four columns was extracted and then examined.

LC separation of the eighteen cannabinoids was first performed using a Poroshell 120 EC-C18 column and 77.5, 75.0, and 70.0% (*v*/*v*) acetonitrile in the mobile phase. Baseline separation of the eight acidic cannabinoids was achieved using 70.0% (*v*/*v*) acetonitrile in the mobile phase, except for the CBLA/CBCA pair, which might indicate that the acidic cannabinoids were not totally neutralized. To fully neutralize the acidic cannabinoids, a stronger acid, e.g., trifluoroacetic acid, at a higher concentration, e.g., 0.1% (*v*/*v*) could be used as the A solvent [20]. However, further experiments were not performed as the obtained dataset already satisfied the requirements by this study. Next, LC-UV separation of the eighteen cannabinoids was carried out using a Raptor ARC-18 column and 77.5, 75.0, and 70.0% (*v*/*v*) acetonitrile in the mobile phase. Baseline separation of the eight acidic cannabinoids was also achieved using 70.0% (*v*/*v*) acetonitrile in the mobile phase, except for the CBLA/CBCA pair. Then, LC-UV separation of the eighteen cannabinoids was evaluated using a Cortecs Shield RP-18 column and 75.0, 70.0, and 65.0% (*v*/*v*) acetonitrile in the mobile phase. Baseline separation of the eight acidic cannabinoids was achieved using 65.0% (*v*/*v*) acetonitrile in the mobile phase. Finally, LC-UV separation of the eighteen cannabinoids was assessed using an Ascentis Express RP-Amide column and 80.0, 77.5, 70.0, and 65.0% (*v*/*v*) acetonitrile in the mobile phase. Baseline separation of the eight acidic cannabinoids was achieved using 70.0% (*v*/*v*) acetonitrile in the mobile phase.

**Figure 2:**
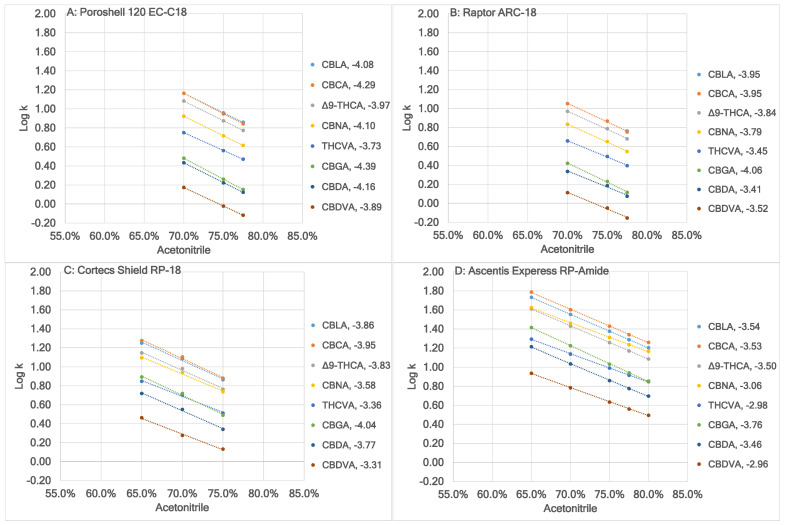
A comparison of Log k of the eight acidic cannabinoids at different acetonitrile content in the mobile phase using four different columns. The A solvent was 0.02% (*v*/*v*) HCO_2_H. The B solvent was acetonitrile. The lowest correlation coefficient of these plots was 0.9991.

Figure 2 shows a comparison of Log k of the eight acidic cannabinoids at different acetonitrile content using the four columns. The retention factor of the eight acidic cannabinoids increased in the flowing order: Raptor ARC-18, Poroshell 120 EC-C18/Cortecs Shield RP-18, and Ascentis Express RP-Amide, which is exemplified in Table 2 when the mobile phase contained 70.0% (*v*/*v*) acetonitrile. Between Poroshell 120 EC-C18 and Cortecs Shield RP-18, higher retention factor of CBDVA, CBDA, CBGA and CBNA was obtained using Cortecs Shield RP-18, while higher retention factor of THCVA, Δ^9^-THCA, CBLA, and CBCA was obtained using Poroshell 120 EC-C18.

The two traditional RP stationary phases resembled each other: (1) the eight acidic cannabinoids with ascending retention factor were in the same sequence (Figure 2A,B); and (2) CBDVA, CBDA, and CBGA had smaller retention factor than their neutral counterparts, while THCVA, CBNA, Δ^9^-THCA, CBLA, and CBCA had larger retention factor than their neutral counterparts (Table 1 and Table 2). The two traditional RP stationary phases differed from each other in the lower selectivity of the CBDA/CBGA pair by Poroshell 120 EC-C18. Using values in Table 2, it was calculated to be 1.115 and 1.223, respectively, for Poroshell 120 EC-C18 and Raptor ARC-18. In addition, the two traditional RP stationary phases differed from each other in the smaller differences in retention factor of the CBNA/CBN and CBCA/CBC pairs by Poroshell 120 EC-C18. Using the values in Table 2, differences in retention factor of the CBNA/CBN and CBCA/CBC pairs were calculated to be 0.81 and 0.63 for Poroshell 120 EC-C18, respectively, and 1.01 and 1.12, respectively, for Raptor ARC-18.

The two polar-embedded RP stationary phases resembled each other in that all eight acidic cannabinoids had larger retention factor than their neutral counterparts (Table 1 and Table 2). The two polar-embedded RP stationary phases also differed from each other: (1) the RP-amide stationary phase had significantly larger differences in the retention factor of an acidic cannabinoid and its neutral counterpart with the same acetonitrile content in the mobile phase than the RP-carbamate stationary phase (Table 1 and Table 2); and (2) the selectivity of the THCVA/CBGA and CBCA/CBLA pair had large differences (Figure 2C,D).

A change in selectivity of the eight acidic cannabinoids between the two traditional RP and two polar-embedded RP stationary phases was obvious, including the reversed elution order of the THCVA/CBGA pair. Overall, the observations supported that the ascending polarity order of the four columns in retention of cannabinoids was as follows: Poroshell 120 EC-C18, Raptor ARC-18, Cortecs Shield RP-18, and Ascentis Express RP-Amide.

### 2.3. Separation of the Eighteen Cannabinoids Altogether by LC-DAD

Once the baseline separation of the ten neutral and eight acidic cannabinoids was proven to be achievable, although individually, further separation optimization continued to separate the eighteen cannabinoids altogether. For each column, the acetonitrile content in the mobile phase was first determined to guarantee baseline separation of the ten neutral cannabinoids. Then, the pH of the A solvent was varied to avoid coelution of any acidic cannabinoids with any neutral cannabinoids, based on the fact that the retentions of acidic cannabinoids were dependent on pH, while the retentions of neutral cannabinoids were not.

Supplementary Figure S2 demonstrates LC-UV chromatograms of the eighteen cannabinoids using a Poroshell 120 EC-C18 column, 70.0% (*v*/*v*) acetonitrile in the mobile phase, and 0.03% HCO_2_H + 0.5 mM NH_4_HCO_2_ (pH 2.97), 0.02% HCO_2_H + 1 mM NH_4_HCO_2_ (pH 3.15), and 0.01% HCO_2_H + 1 mM NH_4_HCO_2_ (pH 3.38) as the A solvent. It can be seen that as the pH of the A solvent increased, the retentions of acidic cannabinoids decreased, but the retentions of neutral cannabinoids did not, resulting in resolution changes in acidic and neutral cannabinoids. Best separation was achieved using 0.02% HCO_2_H + 1 mM NH_4_HCO_2_ (pH 3.15) as the A solvent. However, the resolutions of CBN/CBNA and Δ^9^-THCA/CBC were slightly above the baseline. It could be accepted if better separations were not achieved later. It was noted that this minor problem could not be easily solved as the two acidic cannabinoids were on the opposite sides of the two pairs.

Supplementary Figure S3 demonstrates LC-UV chromatograms of eighteen cannabinoids using a Poroshell 120 EC-C18 column, 75.0% (*v*/*v*) acetonitrile in the mobile phase, and 0.03% HCO_2_H + 0.5 mM NH_4_HCO_2_ (pH 2.97), 0.02% HCO_2_H + 1 mM NH_4_HCO_2_ (pH 3.15), and 0.01% HCO_2_H + 1 mM NH_4_HCO_2_ (pH 3.38) as the A solvent. In comparison with Supplementary Figure S2 at the same pH of the A solvent, the shorter retention times and changes in the eluting order of cannabinoids were attributed to the higher acetonitrile content in the mobile phase. The best separation was achieved using 0.03% HCO_2_H + 0.5 mM NH_4_HCO_2_ (pH 2.97) as the A solvent, but the resolution of CBGA/CBDA/CBG and CBC/Δ^9^-THCA was still slightly above the baseline. This separation (Supplementary Figure S3A) was less acceptable than the previous one (Supplementary Figure S2B) due to lower resolution of the CBC/Δ^9^-THCA pair. Its advantage was a shorter run time. To further achieve baseline separation of all eighteen cannabinoids, two columns could be used sequentially. However, the total run time would be doubled, which could be shortened by using a higher flow rate. This concept was explored using 77.5% (*v*/*v*) acetonitrile due to an even shorter run time and a lower backpressure so that a higher flow rate could be used.

**Figure 3:**
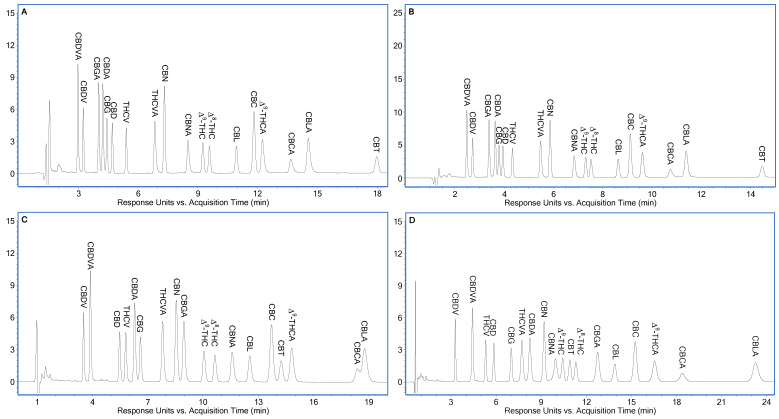
Optimized separations of the eighteen cannabinoids at 1 µg/mL individual concentration for potency testing of hemp-based products by LC-DAD. (**A**) Column: Poroshell 120 EC-C18 2 × 150 mm × 2.1 mm; A solvent: 0.03% HCO_2_H + 0.5 mM NH_4_HCO_2_ (pH 2.97); B solvent: acetonitrile; mobile phase: 77.5% (*v*/*v*) B; flow rate: 0.45 mL/min. (**B**) Column: Raptor ARC-18 2 × 150 mm × 2.1 mm; A solvent: 0.03% HCO_2_H + 0.5 mM NH_4_HCO_2_ (pH 2.97); B solvent: acetonitrile; mobile phase: 75.0% (*v*/*v*) B; flow rate: 0.5 mL/min. (**C**) Column: Cortecs Shield RP-18 150 mm × 2.1 mm; A solvent: 0.02% HCO_2_H + 1 mM NH_4_HCO_2_ (pH 3.15); B solvent: acetonitrile; mobile phase: 65.0% (*v*/*v*) B; flow rate: 0.3 mL/min. (**D**) Column: Ascentis Express RP-Amide 150 mm × 2.1 mm; A solvent: 0.01% (*v*/*v*) HCO_2_H + 4 mM NH_4_HCO_2_ (pH 3.80); B solvent: acetonitrile; mobile phase: 65.0% (*v*/*v*) B; flow rate: 0.5 mL/min. Additional experimental conditions are described in Section 3.3. LC-DAD.

Supplementary Figure S4 demonstrates LC-UV chromatograms of eighteen cannabinoids using a Poroshell 120 EC-C18 column, 77.5% (*v*/*v*) acetonitrile in the mobile phase and 0.03% HCO_2_H + 0.5 mM NH_4_HCO_2_ (pH 2.97), 0.02% HCO_2_H + 1 mM NH_4_HCO_2_ (pH 3.15), and 0.01% HCO_2_H + 1 mM NH_4_HCO_2_ (pH 3.38) as the A solvent. In comparison with Supplementary Figure S3 at the same pH of the A solvent, even shorter retention times of cannabinoids were attained as expected. The best separation was achieved using 0.03% HCO_2_H + 0.5 mM NH_4_HCO_2_ (pH 2.97) as the A solvent. This separation was further improved using two columns consecutively and a flow rate of 0.45 mL/min (the highest flow rate that could be used so that the backpressure was lower than 600 bar, i.e., the backpressure limit of the LC system). Baseline separation of eighteen cannabinoids was obtained, which is shown in Figure 3A.

Inspired by the successful separation shown in Figure 3A, it was determined that 75% (*v*/*v*) acetonitrile in the mobile phase should be used by Raptor ARC-18 for further separation optimization. Supplementary Figure S5 demonstrates LC-UV chromatograms of eighteen cannabinoids using a Raptor ARC-18 column, 75.0% (*v*/*v*) acetonitrile in the mobile phase, and 0.03% HCO_2_H + 0.5 mM NH_4_HCO_2_ (pH 2.97), 0.02% HCO_2_H + 1 mM NH_4_HCO_2_ (pH 3.15), and 0.01% HCO_2_H + 1 mM NH_4_HCO_2_ (pH 3.38) as the A solvent. The best separation was achieved using 0.03% HCO_2_H + 0.5 mM NH_4_HCO_2_ (pH 2.97) as the A solvent. This separation was further improved using two columns sequentially and a flow rate of 0.5 mL/min. Baseline separation of eighteen cannabinoids was obtained except for the CBDA/CBG (R = 1.48) and Δ^9^-/Δ^8^-THC (R = 1.43) pairs, which is shown in Figure 3B.

A comparison of Figure 3A,B showed some interesting details: (1) although the two separations had the same elution order, minor differences in resolutions of adjacent peaks were observed for the CBDA/CBGA/CBD/CBG/THCV, CBN/CBNA, Δ^9^-/Δ^8^-THC, and CBL/CBC/Δ^9^-THCA pairs, which was caused by the minor differences in retention factor and selectivity discussed earlier; (2) better separation of the ten neutral cannabinoids by Poroshell 120 EC-C18 could not be converted into better separation of the eighteen cannabinoids due to the mismatch of retention factor of the eight acidic cannabinoids; and (3) there was a tradeoff between better resolution due to higher separation efficiency of the columns at an optimum flow rate of 0.3 mL/min and shorter run time due to a flow rate higher than 0.3 mL/min.

The eighteen cannabinoids were further separated using a Cortecs Shield RP-18 column, 65.0% (*v*/*v*) acetonitrile in the mobile phase, and 0.03% HCO_2_H + 0.5 mM NH_4_HCO_2_ (pH 2.97) (Supplementary Figure S6A), 0.02% HCO_2_H + 1 mM NH_4_HCO_2_ (pH 3.15) (Figure 3C), and 0.01% HCO_2_H + 1 mM NH_4_HCO_2_ (pH 3.38) (Supplementary Figure S6B) as the A solvent. Best separation was achieved using 0.02% HCO_2_H + 1 mM NH_4_HCO_2_ (pH 3.15) as the A solvent (Figure 3C). Because the CBCA/CBLA pair was not baseline separated, another faster separation to baseline separate them was obtained, which will be described later.

The eighteen cannabinoids were further separated using an Ascentis Express RP-Amide column, 65.0% (*v*/*v*) acetonitrile in the mobile phase, and 0.03% HCO_2_H + 0.5 mM NH_4_HCO_2_ (pH 2.97) (Supplementary Figure S7A), 0.02% HCO_2_H + 1 mM NH_4_HCO_2_ (pH 3.15) (Supplementary Figure S7B), 0.01% HCO_2_H + 1 mM NH_4_HCO_2_ (pH 3.38) (Supplementary Figure S7C), 0.01% HCO_2_H + 2 mM NH_4_HCO_2_ (pH 3.55) (Supplementary Figure S7D), and 0.01% (*v*/*v*) HCO_2_H + 4 mM NH_4_HCO_2_ (pH 3.80) (Figure 3D) as the A solvent. Baseline separation was achieved using 0.01% (*v*/*v*) HCO_2_H + 4 mM NH_4_HCO_2_ (pH 3.80) as the A solvent (Figure 3D), except for the CBNA/Δ^9^-THC (R = 1.43) and CBT/Δ^8^-THC (R = 1.34) pairs. In addition, a closer examination of Figure 3D showed imperfect peak shapes of CBNA and CBCA.

**Figure 4:**
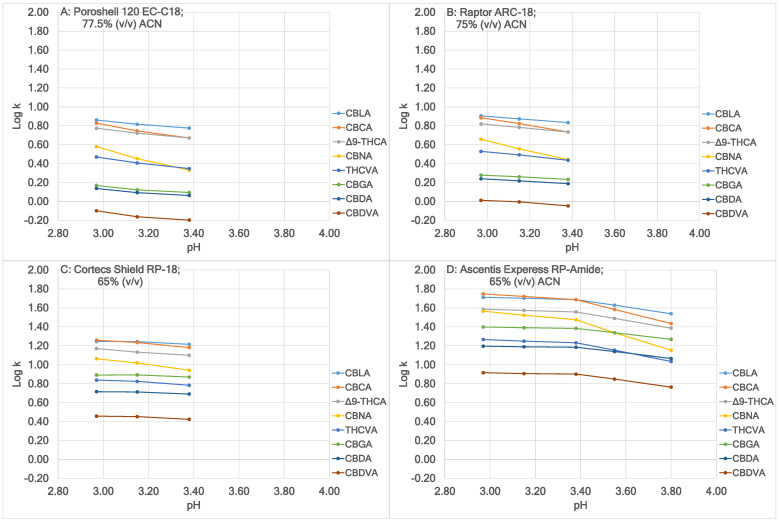
Effect of pH of the A solvent on Log k of the eight acidic cannabinoids using the four columns. Chromatographic conditions were the same as described in Figure 3, except that additional A solvents at additional pHs were included.

Figure 4 shows the effect of pH of the A solvent on Log k of the eight acidic cannabinoids using the four columns. Chromatographic conditions were the same as described in Figure 3, except that additional A solvents at additional pHs were included. It can be seen that as pH increased, the retention factor of all acidic cannabinoids decreased. The effect could be ranked from large to small in the following sequence: CBCA and CBNA, then THCVA, and finally other acidic cannabinoids, which was probably caused by their different acidities. Generally, the imperfection of the peak shapes was in the same order, especially obvious when combined with high retention factors. Figure 4 also demonstrates the dependence of optimized separation on pH stability at pH 2.97, 2.97, 3.15, and 3.80 in Figure 4A, Figure 4B, Figure 4C, and Figure 4D, respectively. While a higher HCO₂H/NH₄HCO₂ buffer concentration resulted in greater pH stability, it also caused increased fluctuations in UV background absorption, leading to a higher LOQ [31]. The optimal buffer concentration is detailed in Figure 3.

### 2.4. Separation of the Eighteen Cannabinoids Altogether by LC-ESI/TOFMS

The optimized separations of eighteen cannabinoids for potency testing of hemp-based products by LC-DAD (Figure 3) could be readily adopted by LC-ESI/MS/MS. However, LC-ESI/MS/MS only requires baseline separation of structural isomers of the eighteen cannabinoids, therefore promising shorter analytical time.

**Figure 5:**
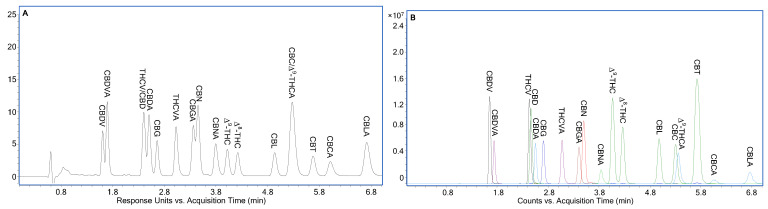
Optimized separation of eighteen cannabinoids at 1 µg/mL individual concentration for potency testing of hemp-based products by LC-ESI/MS/MS. (**A**) LC-UV chromatogram detected at 230 nm; (**B**) LC-ESI/TOFMS EICs of cannabinoids using their [M+H]^+^ ions except [M+H-H_2_O]^+^ ion of CBGA with ±20 ppm. Column: Cortecs Shield RP-18 150 mm × 2.1 mm; A solvent: 0.01% HCO_2_H + 1 mM NH_4_HCO_2_ (pH 3.38); B solvent: acetonitrile; mobile phase: 70.0% (*v*/*v*) B; flow rate: 0.5 mL/min. Additional experimental conditions are described in Section 3.3. LC-DAD and Section 3.4. ESI/TOFMS.

Further separation optimization continued to discover a separation for potency testing of hemp-based products by LC-ESI/MS/MS. It was based on a 10.0 min separation of the ten neutral cannabinoids using a Cortecs Shield RP-18 column and 70.0% (*v*/*v*) acetonitrile in the mobile phase, where all structural isomers were baseline separated despite the coelution of THCV and CBD. Supplementary Figure S8 demonstrates LC-UV chromatograms of the eighteen cannabinoids using 0.03% HCO_2_H + 0.5 mM NH_4_HCO_2_ (pH 2.97), 0.02% HCO_2_H + 1 mM NH_4_HCO_2_ (pH 3.15), and 0.01% HCO_2_H + 1 mM NH_4_HCO_2_ (pH 3.38) as the A solvent. Baseline separation of all structural isomers was achieved using 0.01% HCO_2_H + 1 mM NH_4_HCO_2_ (pH 3.38) as the A solvent within 13.0 min (Supplementary Figure S8C). This separation was further shortened to be within 9.0 and 7.0 min using faster flow rate, i.e., 0.4 (Supplementary Figure S9) and 0.5 mL/min (Figure 5A), respectively, taking advantage of the superior resolution of the Δ^9^-/Δ^8^-THC pairs offered by Cortecs Shield RP-18. Because the CBCA/CBLA pair was separated within 7.0 min in Figure 5A, this separation can be used together with the separation shown in Figure 3C for potency testing of hemp-based products using a Cortecs Shield RP-18 column by LC-DAD. Figure 5B shows the LC-ESI/TOFMS extracted ion chromatograms (EICs) of cannabinoids at 0.5 mL/min using their [M+H]^+^ ions except [M+H-H_2_O]^+^ ion of CBGA with ±20 ppm. Based on this separation, a rapid LC-ESI/MS/MS method using previously reported precursor ions, collision energies, quantifier ions and qualifier ions [21] can be easily developed.

## 3. Materials and Methods

### 3.1. The Chemicals and Reagents

LC grade water (H_2_O, Cat# W5-4 Lot 215301), acetonitrile (MeCN), methanol (MeOH), formic acid (HCO_2_H), and ammonium formate (NH_4_HCO_2_) were purchased from Fisher Scientific (Pittsburgh, PA, USA). All cannabinoid standards were purchased as certified reference materials (CRMs). The majority of cannabinoid standards, including a mixture of CBC, CBD, CBDV, CBG, CBN, Δ^9^-THC, Δ^8^-THC, and THCV at 1 mg/mL in acetonitrile (i.e., Phytocannabinoid Neutrals Mixture 8); two neutral cannabinoids of CBL and CBT at 1 mg/mL in methanol; a mixture of CBC, CBD, CBDA, CBDV, CBG, CBGA, CBN, Δ^9^-THC, Δ^8^-THC, Δ^9^-THCA, and THCV at 250 µg/mL in acetonitrile (i.e., Phytocannabinoid Mixture 11); and four acidic cannabinoids of CBCA, CBDVA, CBNA, and THCVA at 1 mg/mL in acetonitrile were purchased from Cayman Chemical (Ann Arbor, MI, USA). One acidic cannabinoid, i.e., CBLA at 0.5 mg/mL in acetonitrile, was purchased from Millipore Sigma (St. Louis, MO, USA).

### 3.2. Solutions of Standard Cannabinoids

A mixture solution containing ten neutral cannabinoids (i.e., CBC, CBD, CBDV, CBG, CBL, CBN, CBT, Δ^9^-THC, Δ^8^-THC, and THCV) was prepared in methanol at 10 µg/mL individual concentration using Phytocannabinoid Neutrals Mixture 8 and additional individual neutral cannabinoids. Another mixture solution containing ten neutral cannabinoids and eight acidic cannabinoids (i.e., CBC, CBCA, CBD, CBDA, CBDV, CBDVA, CBG, CBGA, CBL, CBLA, CBN, CBNA, CBT, Δ^9^-THC, Δ^9^-THCA, Δ^8^-THC, THCV, and THCVA) was prepared in methanol at 10 µg/mL individual concentration using Phytocannabinoid Mixture 11 and additional individual cannabinoids. The two mixture solutions were diluted 10 times with methanol before each was used during LC separation optimization.

### 3.3. LC-DAD

LC-DAD used an Agilent 1260 Infinity II LC system (Agilent Technologies, Santa Clara, CA, USA), which was equipped with a solvent degasser, binary pump, temperature controlled autosampler, column oven, and DAD.

LC separation used either an Agilent Poroshell 120 EC-C18 column, a Restek (Bellefonte, PA, USA) Raptor ARC-18 column, a Waters (Milford, MA, USA) Cortecs Shield RP-18 column, a Millipore Sigma Ascentis Express RP-Amide column, two sequential Agilent Poroshell 120 EC-C18 columns, or two consecutive Restek Raptor ARC-18 columns. All of the columns had an identical size, i.e., 150 mm × 2.1 mm, and used 2.7 µm core–shell particles. During LC separation, a 0.2 µm ultra-high pressure (UHP) precolumn filter (IDEX Health & Science, Oak Harbor, WA, USA) was installed before the column(s). The column(s) was/were then put inside the column oven at 30 °C.

Mobile phases were mixed with an A solvent and a B solvent by the Agilent 1260 Infinity II LC system. The pH of the mobile phases was controlled by the A solvent using the HCO_2_H/NH_4_HCO_2_ buffer system, including 0.01% (*v*/*v*) HCO_2_H + 4 mM NH_4_HCO_2_ (pH 3.80), 0.01% HCO_2_H + 2 mM NH_4_HCO_2_ (pH 3.55), 0.01% HCO_2_H + 1 mM NH_4_HCO_2_ (pH 3.38), 0.02% HCO_2_H + 1 mM NH_4_HCO_2_ (pH 3.15), 0.03% HCO_2_H + 0.5 mM NH_4_HCO_2_ (pH 2.97), and 0.02% HCO_2_H. The selection of specific concentrations of HCO₂H or NH₄HCO₂ was based on the principles of the Henderson–Hasselbalch equation. The B solvent was acetonitrile.

To maximize the separation efficiency of the columns and resolution of adjacent cannabinoids in the chromatograms, most separation was carried out at a flow rate of 0.3 mL/min. To shorten the run time, separation was also carried out at higher flow rates, e.g., 0.45 and 0.5 mL/min, at the cost of lowered separation efficiency of the columns and decreased resolution of adjacent cannabinoids in the chromatograms.

The autosampler was maintained at 8 °C. The injection volume was 3 μL. Ultraviolet (UV) detection was carried out at 230 nm with 4 nm bandwidth using reference wavelength at 360 nm with 100 nm bandwidth. UV spectra were recorded from 190.0 to 400.0 nm with 2.0 nm step.

### 3.4. ESI/TOFMS

UV detection was followed by ESI/TOFMS detection so that peaks could be verified, especially during coelutions, which significantly improved time efficiency.

ESI/TOFMS used an Agilent 6545 quadrupole time-of-flight (Q-TOF) mass spectrometer that was equipped with a Dual AJS (Agilent Jet Stream) ESI source. ESI was operated in positive ion mode, creating only [M+H]^+^ ions from neutral cannabinoids, but both [M+H]^+^ and [M+H-H_2_O]^+^ ions from acidic cannabinoids. Source dependent conditions were optimized for flow rate at 0.3 mL/min. Compound dependent conditions were optimized for Δ^9^-THC and Δ^9^-THCA by flow injection analysis of 1 µg/mL solution in methanol. Capillary voltage between 1000 and 3500 V with a step of 500 V, nozzle voltage between 0 and 1200 V with a step of 200 V, and fragmentor voltage between 80 and 200 V with a step of 100 V were experimented. Optimized MS conditions were as follows: MS acquisition mass range, 100–1000 *m*/*z*; MS acquisition rate, 5 spectra/s; drying gas temperature, 325 °C; drying gas flow, 10 L/min; nebulizer pressure, 20 psi; sheath gas temperature, 400 °C; sheath gas flow, 12 L/min; capillary voltage, 3000 V; nozzle voltage, 600 V; fragmentor, 120 V; skimmer, 45 V; Oct1 RF Vpp, 750 V; MS reference mass ions, 121.0509, 922.0098.

Under optimized MS conditions, maximum abundance of [M+H]^+^ ions was obtained for both neutral and acidic cannabinoids. In addition, more abundant [M+H]^+^ ions than [M+H-H_2_O]^+^ ions were obtained for all acidic cannabinoids, except for CBGA. Therefore, for each LC-UV chromatogram of cannabinoids, its corresponding LC-ESI/TOFMS EICs were produced using the [M+H-H_2_O]^+^ ions of CBGA and the [M+H]^+^ ions of all other cannabinoids with ±20 ppm. However, unnecessary LC-ESI/TOFMS EICs were not shown.

## 4. Conclusions

For potency testing of hemp-based products by LC-DAD, the two traditional RP stationary phases (Poroshell 120 EC-C18: -OSi(CH_3_)_2_C_18_H_37_; Raptor ARC-18: -OSi(*i*Bu)_2_C_18_H_37_) were the best choices due to their shorter run times. For potency testing of hemp-based products by LC-ESI/MS/MS, the RP-carbamate stationary phase (Cortecs Shield RP-18: -OSi(CH_3_)_2_C_3_H_6_NH(CO)C_15_H_31_) was the best choice. The RP-Amide stationary phase (Ascentis Express RP-Amide: -OSi(CH_3_)_2_C_3_H_6_NH(CO)C_15_H_31_) was the least preferred for potency testing of hemp-based products by either LC-DAD or LC-ESI/MS/MS because some acidic cannabinoids had high retention factor and imperfect peak shapes. The two traditional RP stationary phases resembled each other closely, but small differences in selectivity led to substantial differences in resolution. The two polar-embedded RP stationary phases resembled each other loosely, and considerable differences in retention factor and selectivity led to huge differences in separation. The hydrophobicity of the columns in retention of cannabinoids was ranked in an ascending order of Cortecs Shield RP-18, Raptor ARC-18, Poroshell 120 EC-C18, and Ascentis Express RP-Amide, while the polarity of the columns in retention of cannabinoids was ranked in an ascending order of Poroshell 120 EC-C18, Raptor ARC-18, Cortecs Shield RP-18, and Ascentis Express RP-Amide. The polarity differences had significant effects on the retention factor and selectivity of CBG/CBD/THCV, Δ^9^-/Δ^8^-THC, CBC/CBT, and most acidic cannabinoids.

Due to content limitations within a single article, additional findings from this study on method validation, sample analysis, and matrix effects will be published separately. However, these procedures can be readily performed by following our recently published methods [19,20,21,31]. In these studies, LC separation of cannabinoids were also optimized at an individual concentration of 1 µg/mL, with subsequent method validation complying with the ISO 17025 guidelines, while sample analysis successfully met the regulatory standards established by the AOAC International [9,10,12]. Furthermore, method specificity was assessed using ESI/TOFMS following UV detection, confirming the absence of false-positive cannabinoid identification caused by matrix effects in edibles, as well as minimal interference from unknown cannabinoids in hemp plant materials at LOQ levels. Moving forward, a challenge in the field is the separation of synthetic THC isomers, including Δ**^8^**-THC, Δ^9^-THC, Δ^9,11^-THC, (6aR, 9R)-Δ^10^-THC, (6aR, 9S)-Δ^10^-THC, and Δ^6a,10a^-THC. Future studies may benefit from incorporating newly developed stationary phases [32,33].

## Figures and Tables

**Table 1:** Retention factors of the ten neutral cannabinoids using 70.0% (*v*/*v*) acetonitrile in the mobile phase. The A solvent was 0.02% (*v*/*v*) HCO_2_H.

Column	Cortecs	Raptor	Poroshell	Ascentis
CBDV	1.68	1.84	2.22	2.91
CBG	3.43	3.30	3.96	6.49
CBD	3.01	3.47	4.24	5.44
THCV	3.01	3.72	4.76	5.19
CBN	4.75	5.79	7.56	8.97
Δ^9^-THC	5.70	7.60	10.05	10.25
Δ^8^-THC	6.04	7.94	10.56	11.11
CBL	7.24	9.30	12.28	13.91
CBC	7.77	10.15	13.95	14.88
CBT	8.51	16.43	21.12	10.80

**Table 2:** Retention factors of the eight acidic cannabinoids using 70.0% (*v*/*v*) acetonitrile in the mobile phase. The A solvent was 0.02% (*v*/*v*) HCO_2_H.

Column	Cortecs	Raptor	Poroshell	Ascentis
CBDVA	1.89	1.30	1.49	6.07
CBDA	3.55	2.16	2.72	10.83
CBGA	5.23	2.65	3.03	16.75
THCVA	5.03	4.55	5.62	13.72
CBNA	8.70	6.80	8.37	28.81
Δ^9^-THCA	9.58	9.33	12.09	26.89
CBLA	12.20	11.27	14.58	35.66
CBCA	12.71	11.27	14.58	39.93

## Data Availability

The datasets generated during and/or analyzed during the current study are available from the corresponding author upon reasonable request.

## Supplementary Materials

The following supporting information can be downloaded at: https://www.mdpi.com/article/10.3390/molecules30040758/s1. Figure S1. Chemical structure of the eighteen cannabinoids for potency testing of hemp-based products (neutral cannabinoids are marked by bold letters). Figure S2. LC separation of the eighteen cannabinoids using Poroshell 120 EC-C18: effect of the pH of the A solvent on the separation. The A solvent was (A) 0.03% HCO_2_H + 0.5 mM NH_4_HCO_2_ (pH 2.97), (B) 0.02% HCO_2_H + 1 mM NH_4_HCO_2_ (pH 3.15), and (C) 0.01% HCO_2_H + 1 mM NH_4_HCO_2_ (pH 3.38); the B solvent was acetonitrile; the mobile phase contained 70.0% (*v*/*v*) B; the flow rate was 0.3 mL/min; and the eighteen cannabinoids were at 1 µg/mL individual concentration. Figure S3. LC separation of the eighteen cannabinoids using Poroshell 120 EC-C18: effect of the pH of the A solvent on the separation. The A solvent was (A) 0.03% HCO_2_H + 0.5 mM NH_4_HCO_2_ (pH 2.97), (B) 0.02% HCO_2_H + 1 mM NH_4_HCO_2_ (pH 3.15), and (C) 0.01% HCO_2_H + 1 mM NH_4_HCO_2_ (pH 3.38); the B solvent was acetonitrile; the mobile phase contained 75.0% (*v*/*v*) B; the flow rate was 0.3 mL/min; and the eighteen cannabinoids were at 1 µg/mL individual concentration. Figure S4. LC separation of the eighteen cannabinoids using Poroshell 120 EC-C18: effect of the pH of the A solvent on the separation. The A solvent was (A) 0.03% HCO_2_H + 0.5 mM NH_4_HCO_2_ (pH 2.97), (B) 0.02% HCO_2_H + 1 mM NH_4_HCO_2_ (pH 3.15), and (C) 0.01% HCO_2_H + 1 mM NH_4_HCO_2_ (pH 3.38); the B solvent was acetonitrile, the mobile phase contained 77.5% (*v*/*v*) B; the flow rate was 0.3 mL/min; and the eighteen cannabinoids were at 1 µg/mL individual concentration. Figure S5. LC separation of eighteen cannabinoids using Raptor ARC-18: effect of the pH of the A solvent on the separation. The A solvent was (A) 0.03% HCO_2_H + 0.5 mM NH_4_HCO_2_ (pH 2.97), (B) 0.02% HCO_2_H + 1 mM NH_4_HCO_2_ (pH 3.15), and (C) 0.01% HCO_2_H + 1 mM NH_4_HCO_2_ (pH 3.38); the B solvent was acetonitrile; the mobile phase contained 75.0% (*v*/*v*) B; the flow rate was 0.3 mL/min; the eighteen cannabinoids were at 1 µg/mL individual concentration. Figure S6. LC separation of eighteen cannabinoids using Cortecs Shield RP-18: effect of the pH of the A solvent on the separation. The A solvent was (A) 0.03% HCO_2_H + 0.5 mM NH_4_HCO_2_ (pH 2.97), and (B) 0.01% HCO_2_H + 1 mM NH_4_HCO_2_ (pH 3.38); the B solvent was acetonitrile; the mobile phase contained 65.0% (*v*/*v*) B, the flow rate was 0.3 mL/min; the eighteen cannabinoids were at 1 µg/mL individual concentration. Figure S7. LC separation of eighteen cannabinoids using Ascentis Express RP-Amide: effect of the pH of the A solvent on the separation. The A solvent was (A) 0.03% HCO_2_H + 0.5 mM NH_4_HCO_2_ (pH 2.97), (B) 0.02% HCO_2_H + 1 mM NH_4_HCO_2_ (pH 3.15), (C) 0.01% HCO_2_H + 1 mM NH_4_HCO_2_ (pH 3.38), and (D) 0.01% HCO_2_H + 2 mM NH_4_HCO_2_ (pH 3.55); the B solvent was acetonitrile; the mobile phase contained 65.0% (*v*/*v*) B; the flow rate was 0.5 mL/min; the eighteen cannabinoids were at 1 µg/mL individual concentration. Figure S8. LC separation of the eighteen cannabinoids using Cortecs Shield RP-18: effect of the pH of the A solvent on the separation. The A solvent was (A) 0.03% HCO_2_H + 0.5 mM NH_4_HCO_2_ (pH 2.97), (B) 0.02% HCO_2_H + 1 mM NH_4_HCO_2_ (pH 3.15), (C) 0.01% HCO_2_H + 1 mM NH_4_HCO_2_ (pH 3.38); the B solvent was acetonitrile; the mobile phase contained 70.0% (*v*/*v*) B; the flow rate was 0.3 mL/min; the eighteen cannabinoids were at 1 µg/mL individual concentration. Figure S9. LC separation of eighteen cannabinoids using Cortecs Shield RP-18. The A solvent was 0.01% HCO_2_H + 1 mM NH_4_HCO_2_ (pH 3.38); the B solvent was acetonitrile; the mobile phase contained 70.0% (*v*/*v*) B; the flow rate was 0.4 mL/min; the eighteen cannabinoids were at 1 µg/mL individual concentration. Table S1. Published LC-DAD methods that were able to quantify fourteen and more cannabinoids for potency testing of hemp-based products (neutral cannabinoids are in bold font). Table S2. Published LC-ESI/MS/MS methods that were able to quantify fourteen and more cannabinoids for potency testing of hemp-based products (neutral cannabinoids are in bold font). Table S3. Published LC-ESI/MS methods that were able to quantify fourteen and more cannabinoids for potency testing of hemp-based products (neutral cannabinoids are in bold font). Table S4. Theoretical plate numbers (N) of the ten neutral cannabinoids using 70.0% (*v*/*v*) acetonitrile in the mobile phase. The A solvent was 0.02% (*v*/*v*) HCO_2_H.
