# Supplementary material for: A Systematic Study of Liquid Chromatography in Search of the Best Separation of Cannabinoids for Potency Testing of Hemp-Based Products
Source: Molecules. 2025 Feb 7;30(4):758. doi: 10.3390/molecules30040758 (PMC11858520; doi:10.3390/molecules30040758)
Supplement: Supplementary file 1 [file molecules-30-00758-s001.zip › molecules-3419419-supplementary.pdf]

Supplementary Fig. S1. Chemical structure of the eighteen cannabinoids for potency testing of hemp-based products (neutral cannabinoids are marked by bold letters).

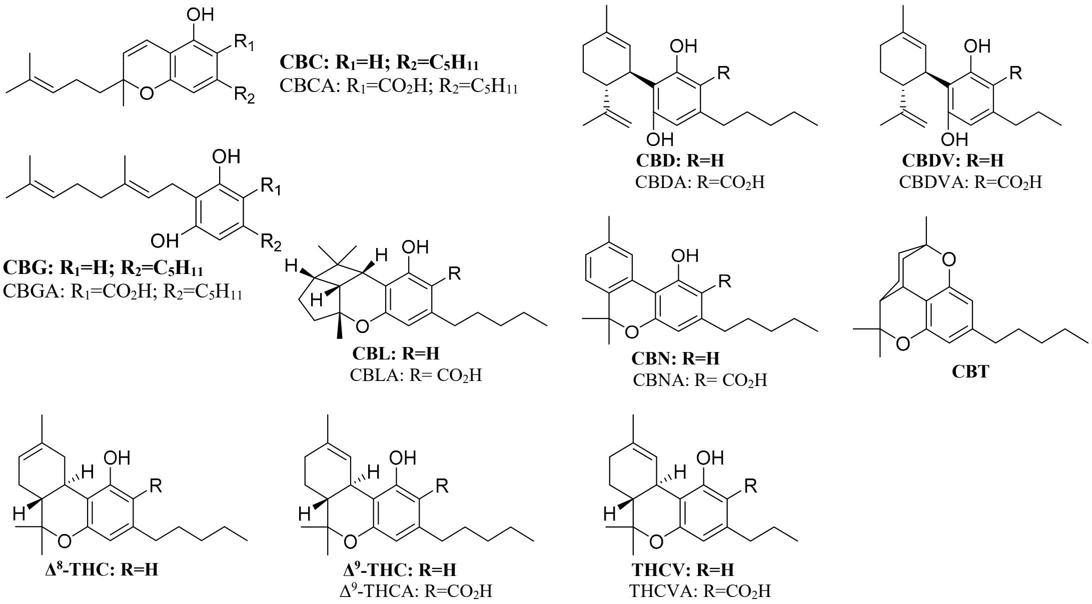

Supplementary Fig. S2. LC separation of the eighteen cannabinoids using Poroshell 120 EC-C18: effect of the pH of the A solvent on the separation. The A solvent was (A) 0.03% HCO_2H + 0.5 mM NH_4HCO_2 (pH 2.97), (B) 0.02% HCO_2H + 1 mM NH_4HCO_2 (pH 3.15), and (C) 0.01% HCO_2H + 1 mM NH_4HCO_2 (pH 3.38); the B solvent was acetonitrile; the mobile phase contained **70.0% (v/v) B**; the flow rate was 0.3 mL/min; and the eighteen cannabinoids were at 1 µg/mL individual concentration.

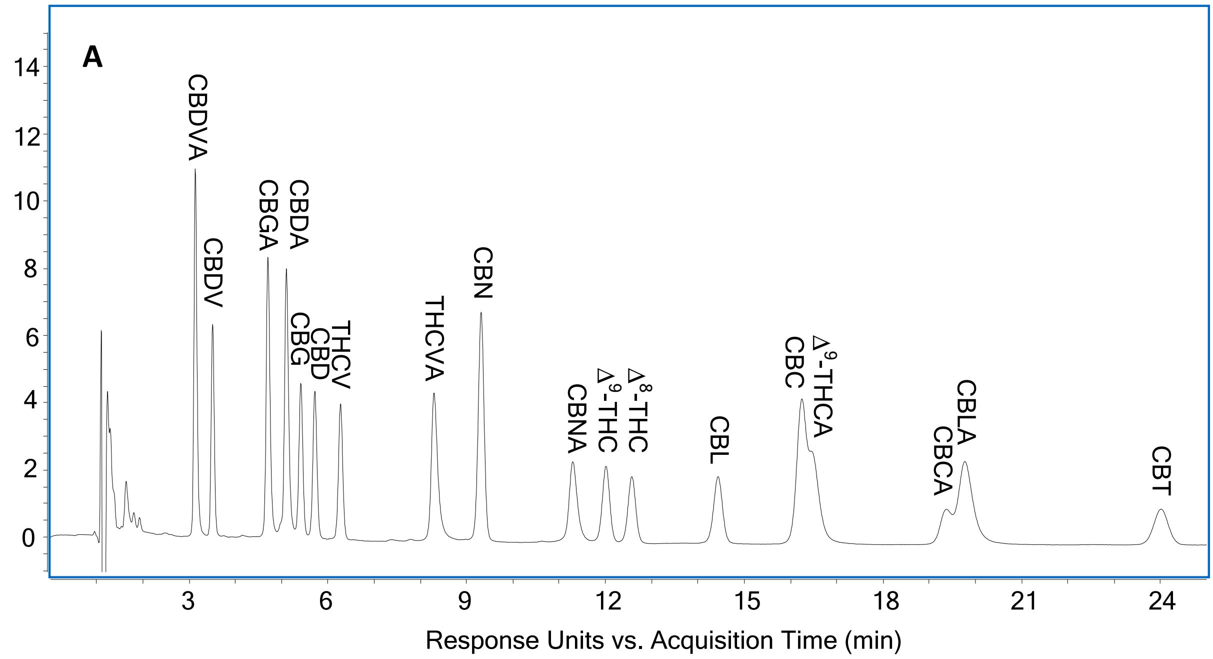

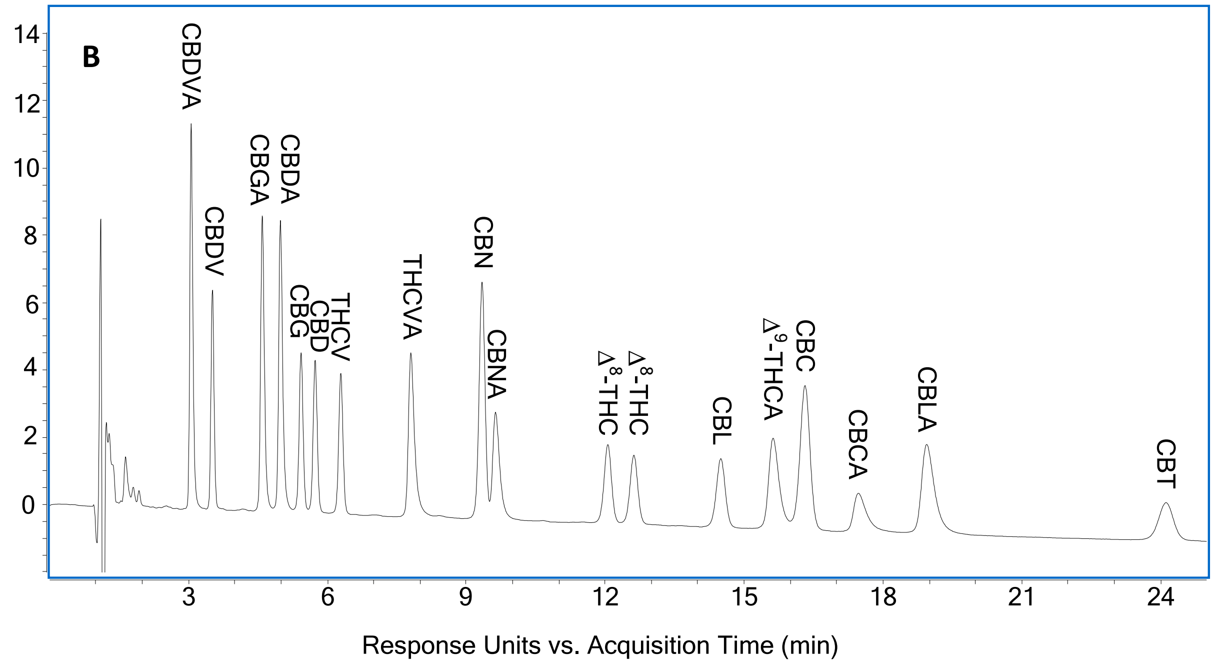
B
CBDVA
CBDV
CBGA
CBDA
CBG
CBD
THCV
THCVA
CBN
CBNA
Δ8-THC
Δ8-THC
CBL
Δ9-THCA
CBC
CBCA
CBLA
CBT
0
2
4
6
8
10
12
14
3
6
9
12
15
18
21
24
Response Units vs. Acquisition Time (min)

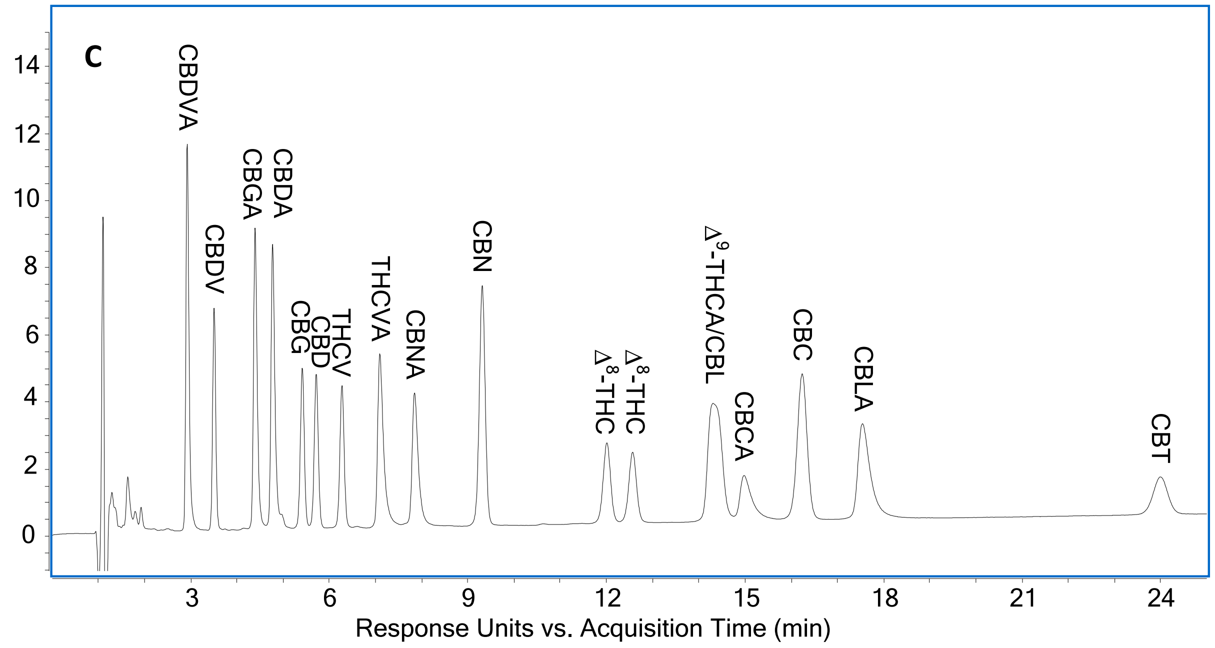
C
CBDVA
CBDV
CBGA
CBDA
CBG
CBD
THCV
THCVA
CBNA
CBN
Δ8-THC
Δ8-THC
Δ9-THCA/CBL
CBCA
CBC
CBLA
CBT
0
2
4
6
8
10
12
14
3
6
9
12
15
18
21
24
Response Units vs. Acquisition Time (min)

Supplementary Fig. S3. LC separation of the eighteen cannabinoids using Poroshell 120 EC-C18: effect of the pH of the A solvent on the separation. The A solvent was (A) 0.03% HCO_2H + 0.5 mM NH_4HCO_2 (pH 2.97), (B) 0.02% HCO_2H + 1 mM NH_4HCO_2 (pH 3.15), and (C) 0.01% HCO_2H + 1 mM NH_4HCO_2 (pH 3.38); the B solvent was acetonitrile; the mobile phase contained **75.0% (v/v) B**; the flow rate was 0.3 mL/min; and the eighteen cannabinoids were at 1 μg/mL individual concentration.

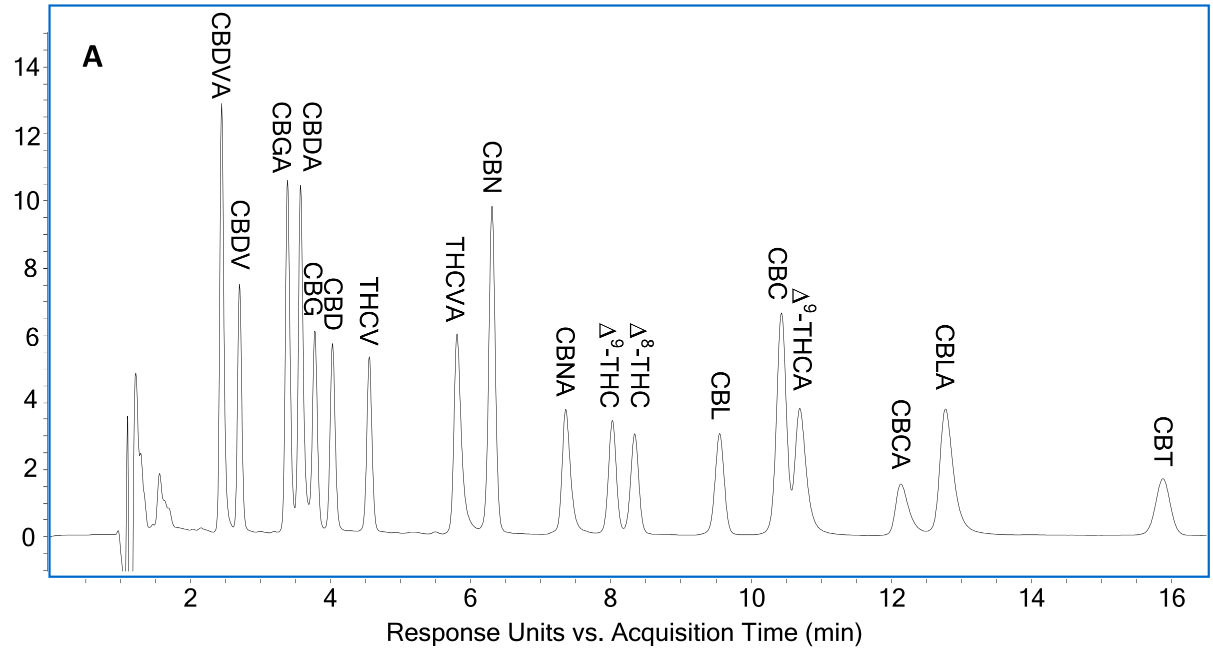

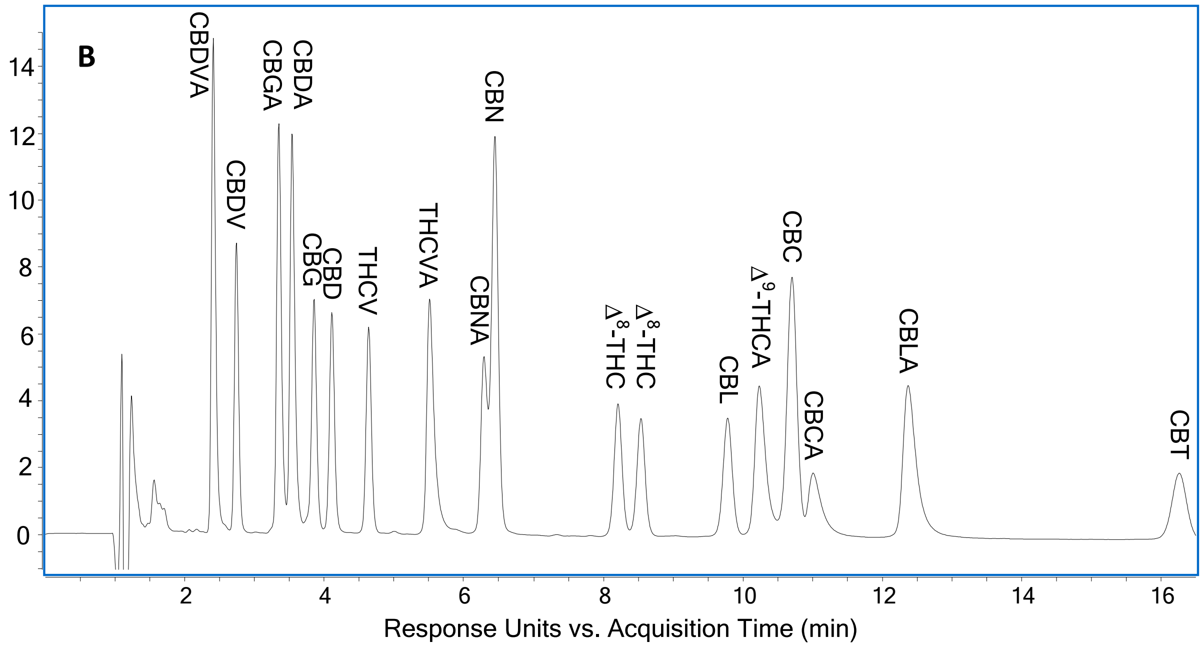
B
CBDVA
CBDV
CBGA
CBDA
CBG
CBD
THCV
THCVA
CBNA
CBN
Δ8-THC
Δ8-THC
CBL
Δ9-THCA
CBC
CBCA
CBLA
CBT
0 2 4 6 8 10 12 14
2 4 6 8 10 12 14 16
Response Units vs. Acquisition Time (min)

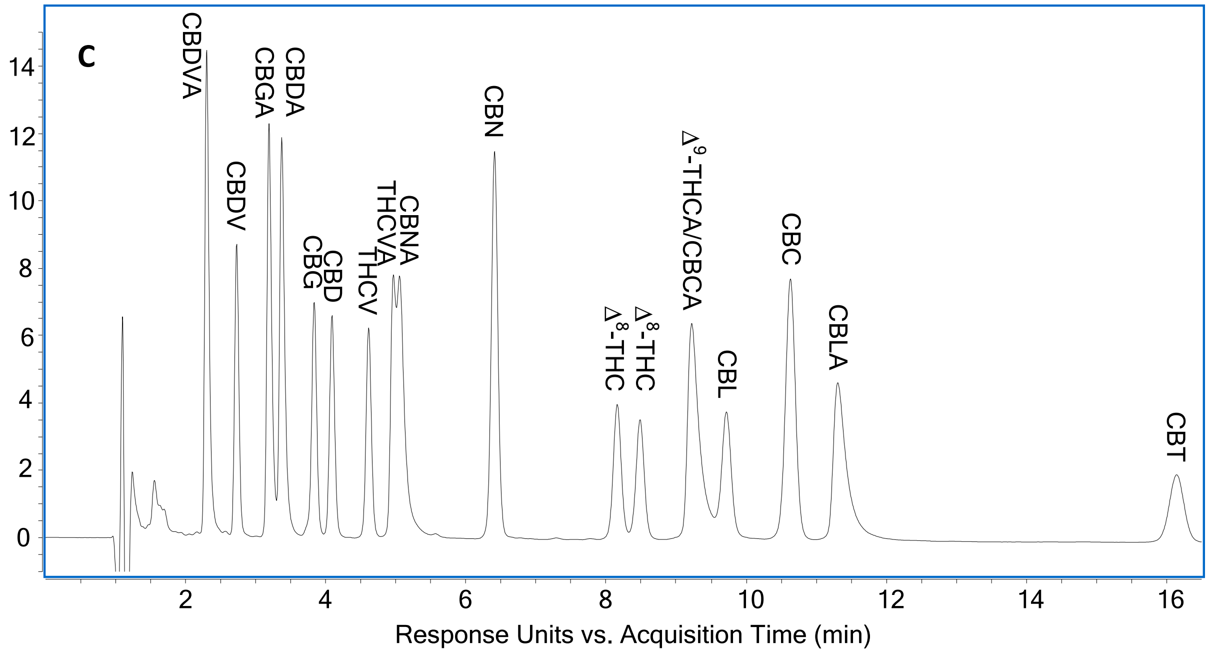
C
CBDVA
CBDV
CBGA
CBDA
CBG
CBD
THCV
THCVA
CBNA
CBN
Δ8-THC
Δ8-THC
Δ9-THCA/CBCA
CBL
CBC
CBLA
CBT
0 2 4 6 8 10 12 14
2 4 6 8 10 12 14 16
Response Units vs. Acquisition Time (min)

Supplementary Fig. S4. LC separation of the eighteen cannabinoids using Poroshell 120 EC-C18: effect of the pH of the A solvent on the separation. The A solvent was (A) 0.03% HCO_2H + 0.5 mM NH_4HCO_2 (pH 2.97), (B) 0.02% HCO_2H + 1 mM NH_4HCO_2 (pH 3.15), and (C) 0.01% HCO_2H + 1 mM NH_4HCO_2 (pH 3.38); the B solvent was acetonitrile, the mobile phase contained **77.5% (v/v) B**; the flow rate was 0.3 mL/min; and the eighteen cannabinoids were at 1 µg/mL individual concentration.

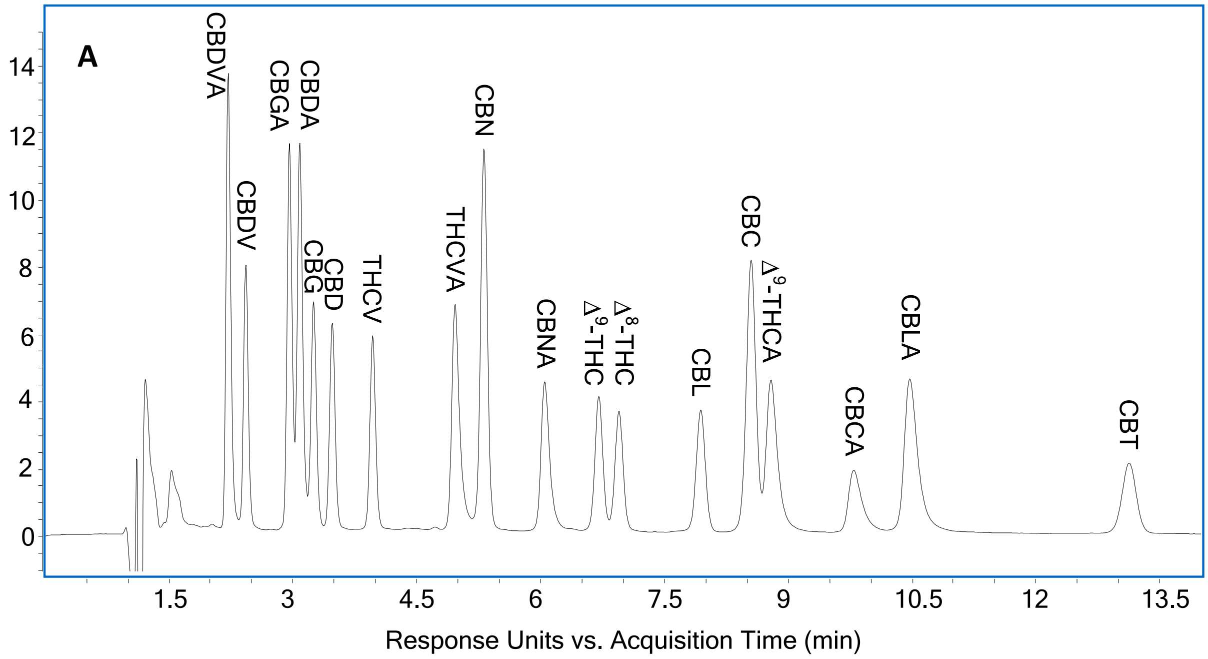

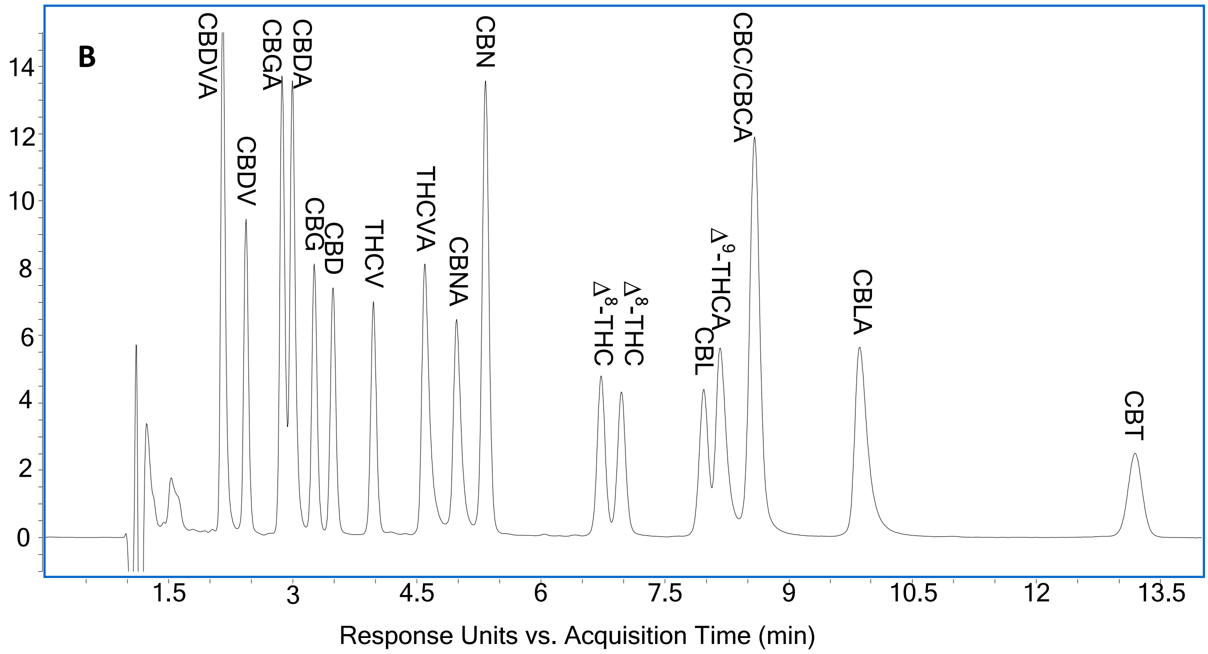
B
CBDVA
CBDV
CBGA
CBDA
CBG
CBD
THCV
THCVA
CBNA
CBN
Δ^8-THC
Δ^8-THC
CBL
Δ^9-THCA
CBC/CBCA
CBLA
CBT
0
2
4
6
8
10
12
14
1.5
3
4.5
6
7.5
9
10.5
12
13.5
Response Units vs. Acquisition Time (min)

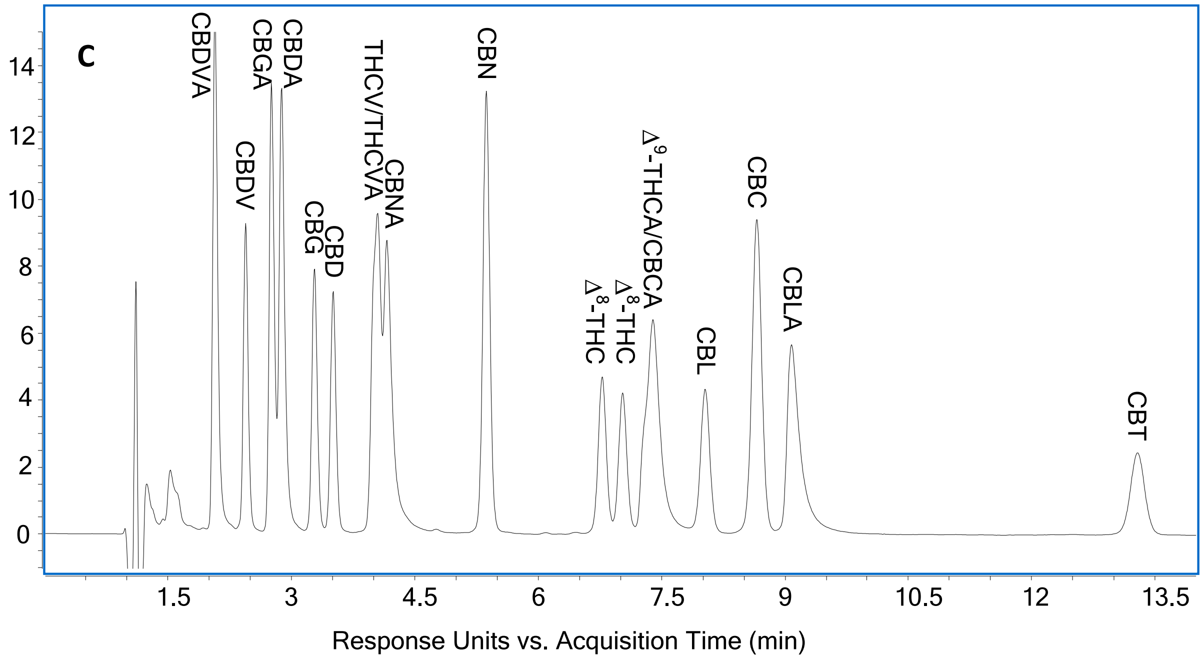
C
CBDVA
CBDV
CBGA
CBDA
CBG
CBD
THCV/THCVA
CBNA
CBN
Δ^8-THC
Δ^8-THC
Δ^9-THCA/CBCA
CBL
CBC
CBLA
CBT
0
2
4
6
8
10
12
14
1.5
3
4.5
6
7.5
9
10.5
12
13.5
Response Units vs. Acquisition Time (min)

Supplementary Fig. S5. LC separation of eighteen cannabinoids using Raptor ARC-18: effect of the pH of the A solvent on the separation. The A solvent was (A) 0.03% HCO_2H + 0.5 mM NH_4HCO_2 (pH 2.97), (B) 0.02% HCO_2H + 1 mM NH_4HCO_2 (pH 3.15), and (C) 0.01% HCO_2H + 1 mM NH_4HCO_2 (pH 3.38); the B solvent was acetonitrile; the mobile phase contained 75.0% (v/v) B; the flow rate was 0.3 mL/min; the eighteen cannabinoids were at 1 µg/mL individual concentration.

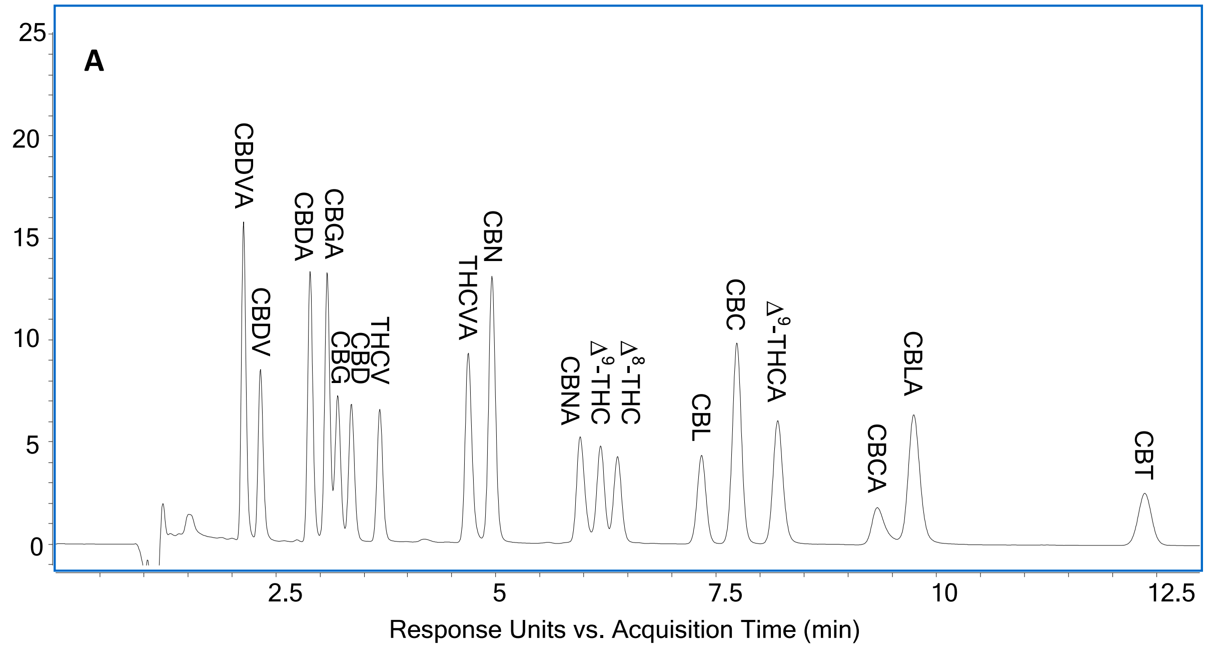

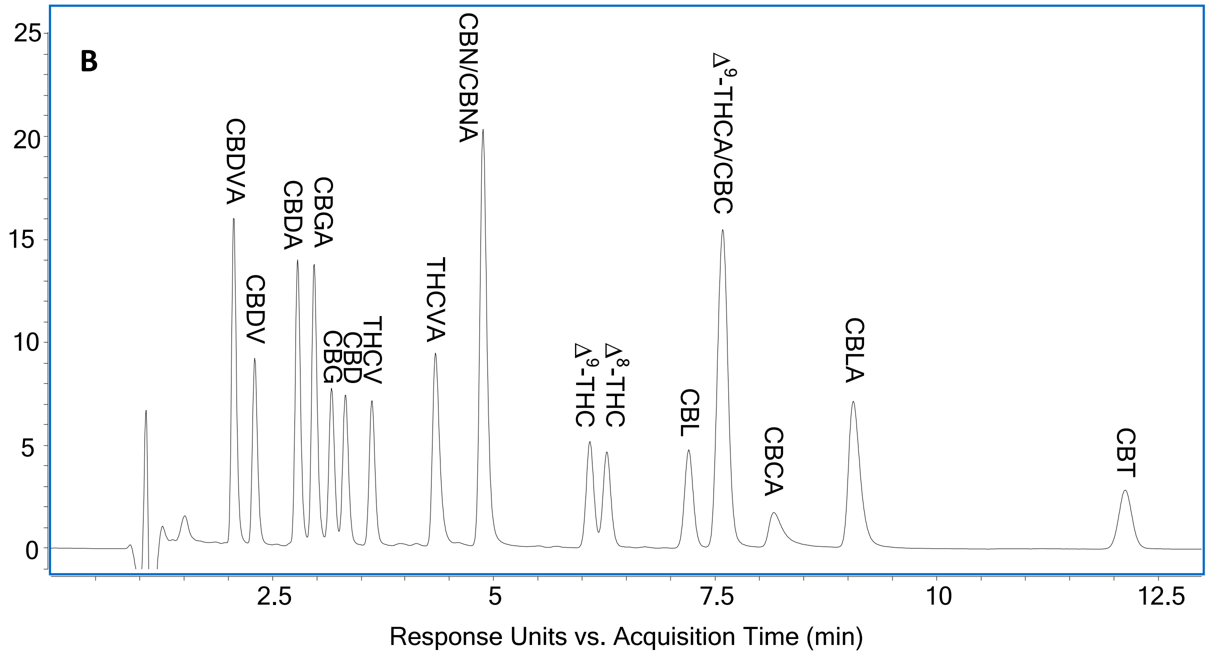
B
CBDVA
CBDV
CBDA
CBGA
CBG
CBD
THCV
THCVA
CBN/CBNA
Δ9-THC
Δ8-THC
CBL
Δ9-THCA/CBC
CBCA
CBLA
CBT
0
5
10
15
20
25
2.5
5
7.5
10
12.5
Response Units vs. Acquisition Time (min)

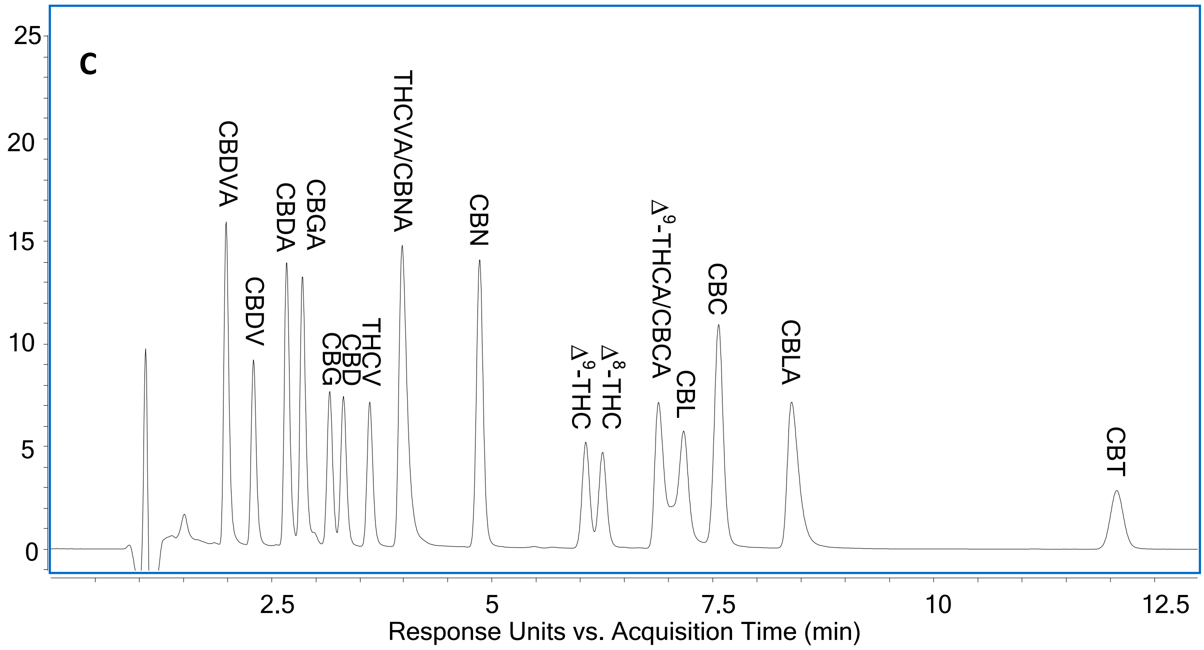
C
CBDVA
CBDV
CBDA
CBGA
CBG
CBD
THCV
THCVA/CBNA
CBN
Δ9-THC
Δ8-THC
Δ9-THCA/CBCA
CBL
CBC
CBLA
CBT
0
5
10
15
20
25
2.5
5
7.5
10
12.5
Response Units vs. Acquisition Time (min)

Supplementary Fig. S6. LC separation of eighteen cannabinoids using Cortecs Shield RP-18: effect of the pH of the A solvent on the separation. The A solvent was (A) 0.03% HCO_2H + 0.5 mM NH_4HCO_2 (pH 2.97), and (B) 0.01% HCO_2H + 1 mM NH_4HCO_2 (pH 3.38); the B solvent was acetonitrile; the mobile phase contained 65.0% (v/v) B, the flow rate was 0.3 mL/min; the eighteen cannabinoids were at 1 μg/mL individual concentration.

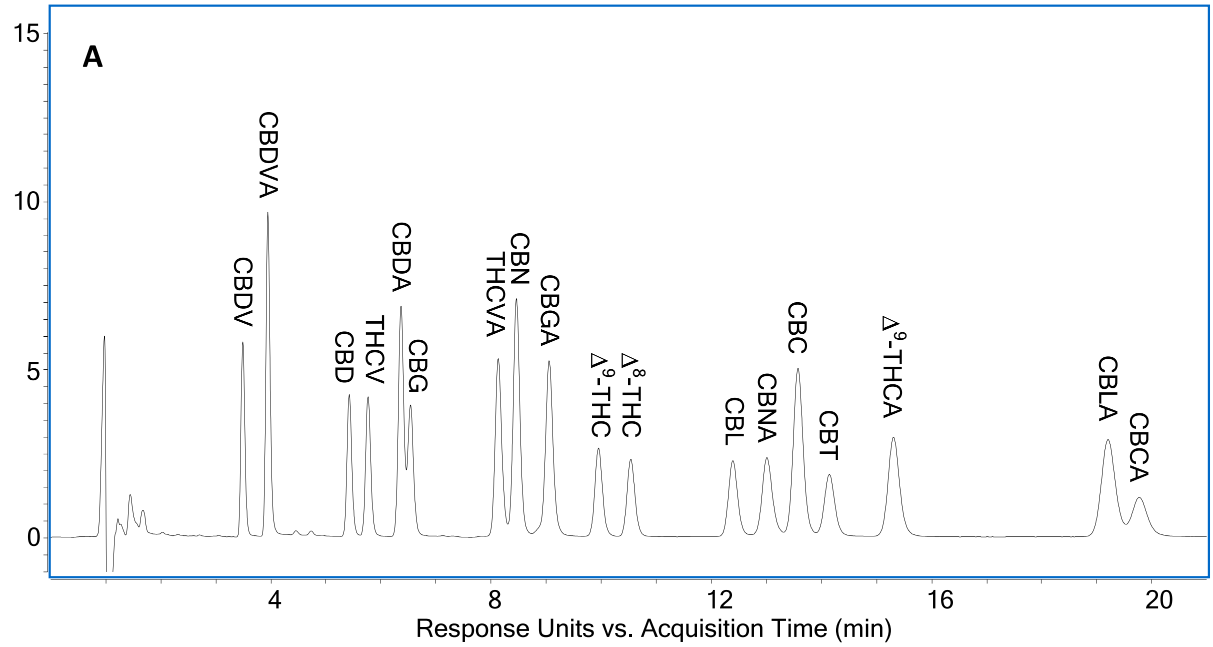

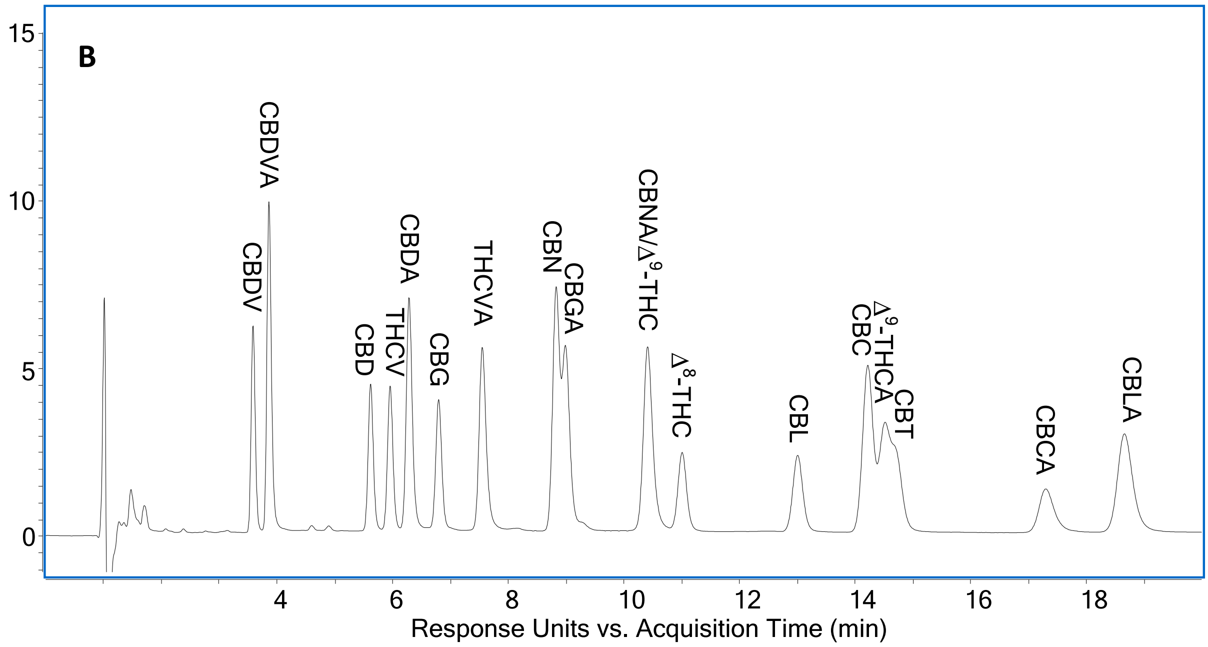
B
15
10
5
0
CBDVA
CBDV
CBDA
THCV
CBD
CBG
THCVA
CBN
CBGA
CBNA/Δ9-THC
Δ8-THC
CBL
Δ9-THCA
CBC
CBT
CBCA
CBLA
4
6
8
10
12
14
16
18
Response Units vs. Acquisition Time (min)

Supplementary Fig. S7. LC separation of eighteen cannabinoids using Ascentis Express RP-Amide: effect of the pH of the A solvent on the separation. The A solvent was (A) 0.03% HCO_2H + 0.5 mM NH_4HCO_2 (pH 2.97), (B) 0.02% HCO_2H + 1 mM NH_4HCO_2 (pH 3.15), (C) 0.01% HCO_2H + 1 mM NH_4HCO_2 (pH 3.38), and (D) 0.01% HCO_2H + 2 mM NH_4HCO_2 (pH 3.55); the B solvent was acetonitrile; the mobile phase contained 65.0% (v/v) B; the flow rate was 0.5 mL/min; the eighteen cannabinoids were at 1 μg/mL individual concentration.

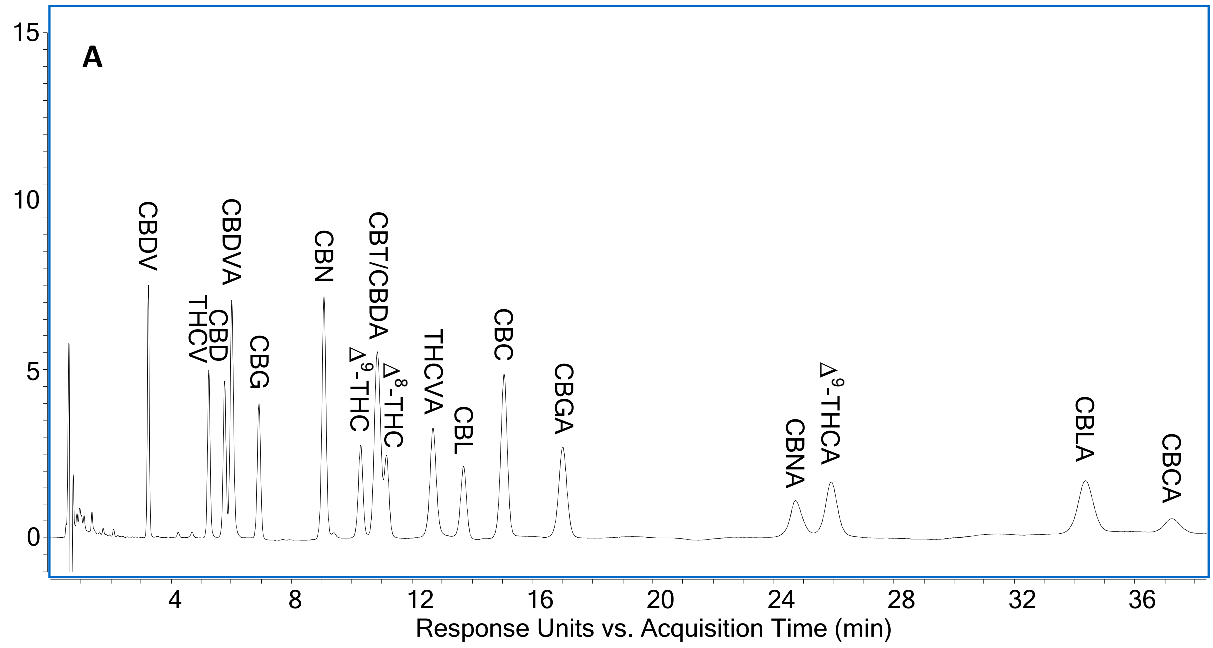

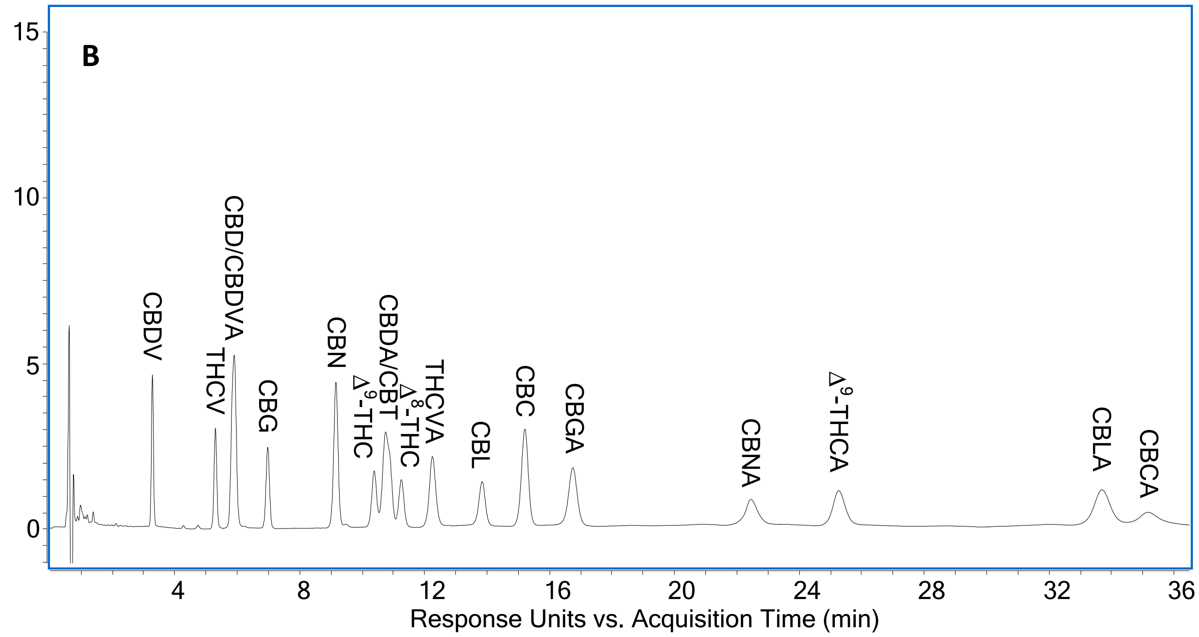
B
15
10
5
0
CBDV
THCV
CBD/CBDVA
CBG
CBN
Δ9-THC
CBDA/CBT
Δ8-THC
THCVA
CBL
CBC
CBGA
CBNA
Δ9-THCA
CBLA
CBCA
4
8
12
16
20
24
28
32
36
Response Units vs. Acquisition Time (min)

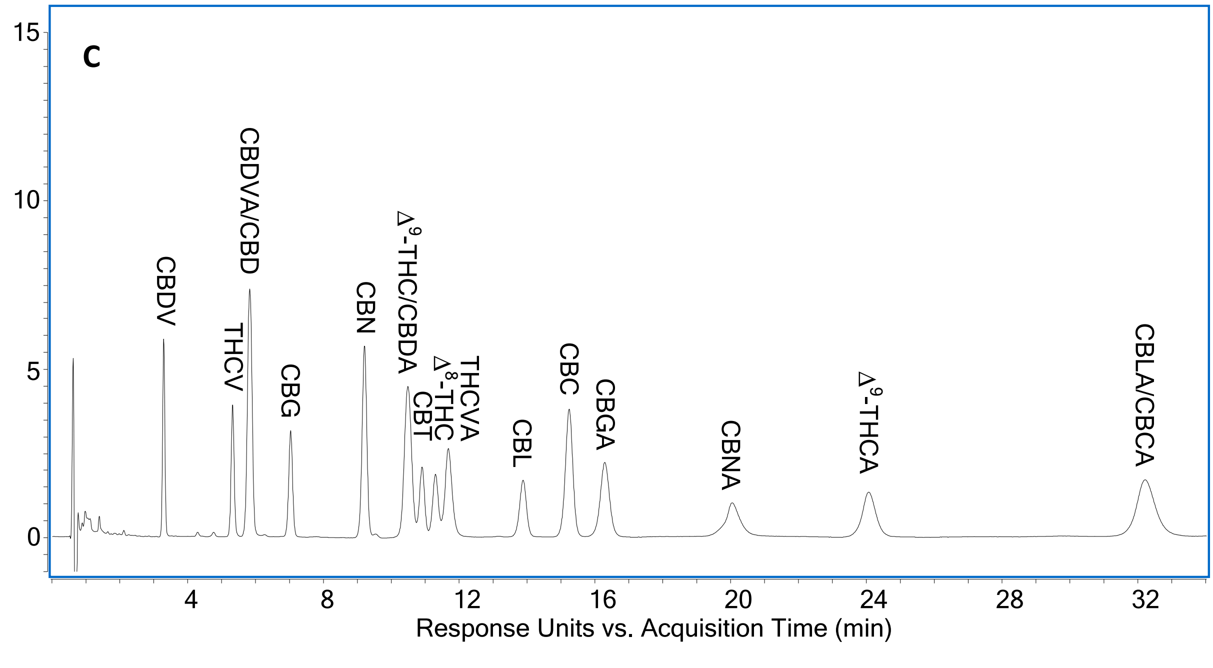
C
15
10
5
0
CBDV
THCV
CBDVA/CBD
CBG
CBN
Δ9-THC/CBDA
CBT
Δ8-THC
THCVA
CBL
CBC
CBGA
CBNA
Δ9-THCA
CBLA/CBCA
4
8
12
16
20
24
28
32
Response Units vs. Acquisition Time (min)

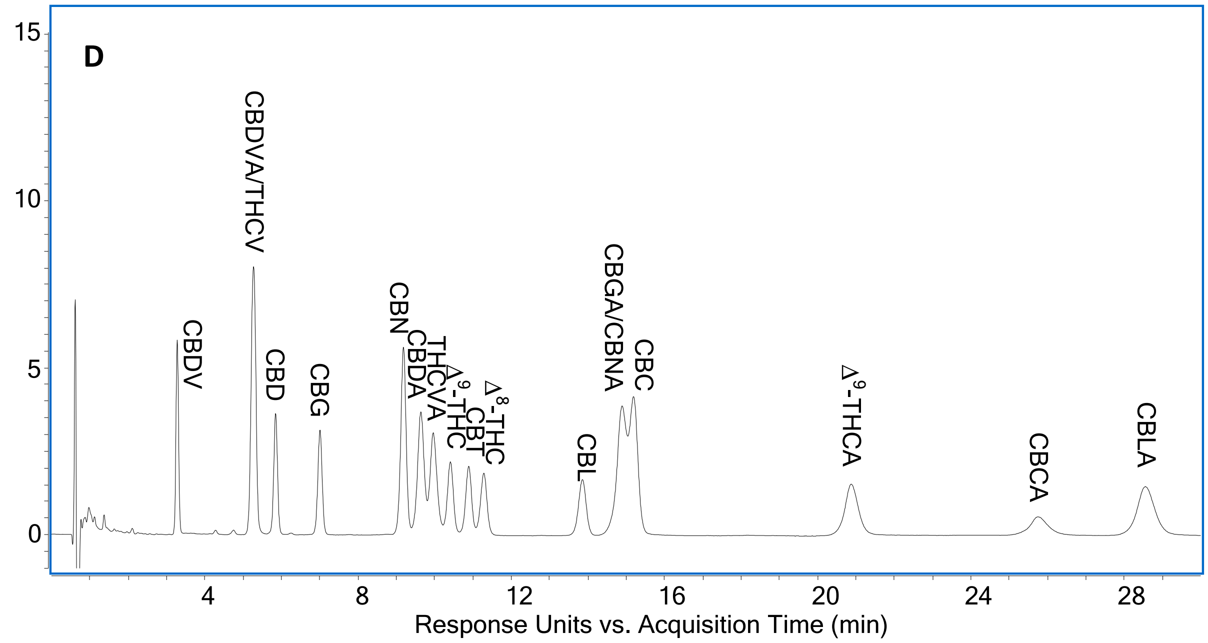
D
CBDV
CBDVA/THCV
CBD
CBG
CBN
CBDA
THCVA
Δ9-THC
CBT
Δ8-THC
CBL
CBGA/CBNA
CBC
Δ9-THCA
CBCA
CBLA
0
5
10
15
4
8
12
16
20
24
28
Response Units vs. Acquisition Time (min)

Supplementary Fig. S8. LC separation of the eighteen cannabinoids using Cortecs Shield RP-18: effect of the pH of the A solvent on the separation. The A solvent was (A) 0.03% HCO_2H + 0.5 mM NH_4HCO_2 (pH 2.97), (B) 0.02% HCO_2H + 1 mM NH_4HCO_2 (pH 3.15), (C) 0.01% HCO_2H + 1 mM NH_4HCO_2 (pH 3.38); the B solvent was acetonitrile; the mobile phase contained 70.0% (v/v) B; the flow rate was 0.3 mL/min; the eighteen cannabinoids were at 1 μg/mL individual concentration.

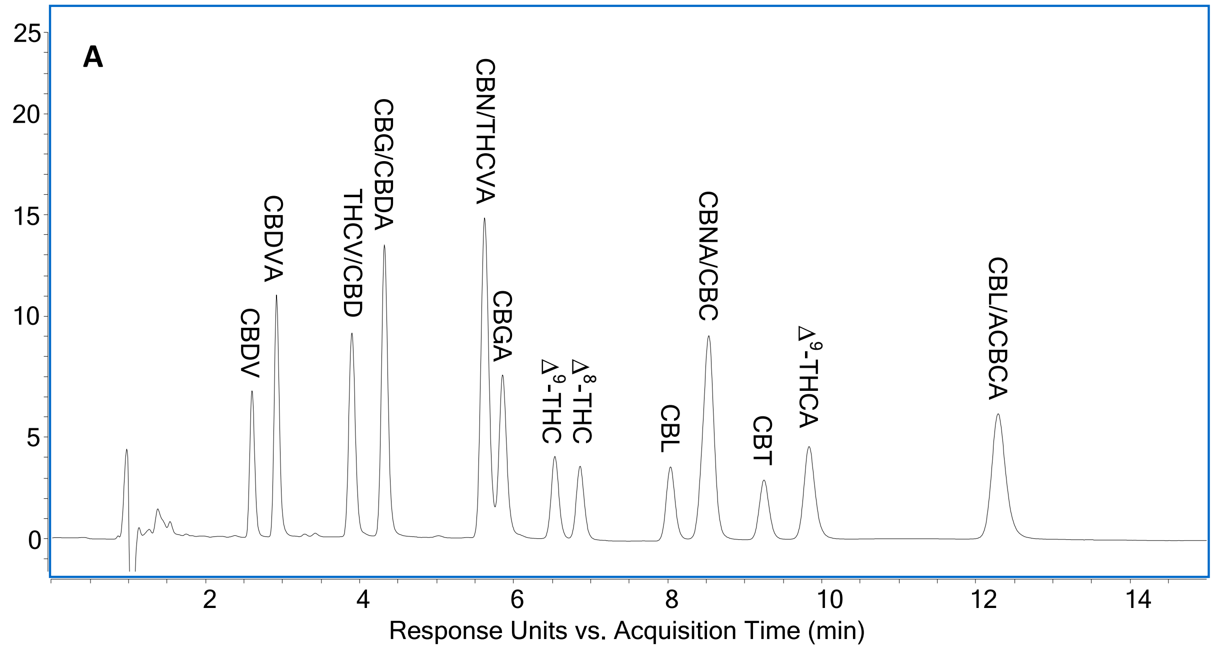

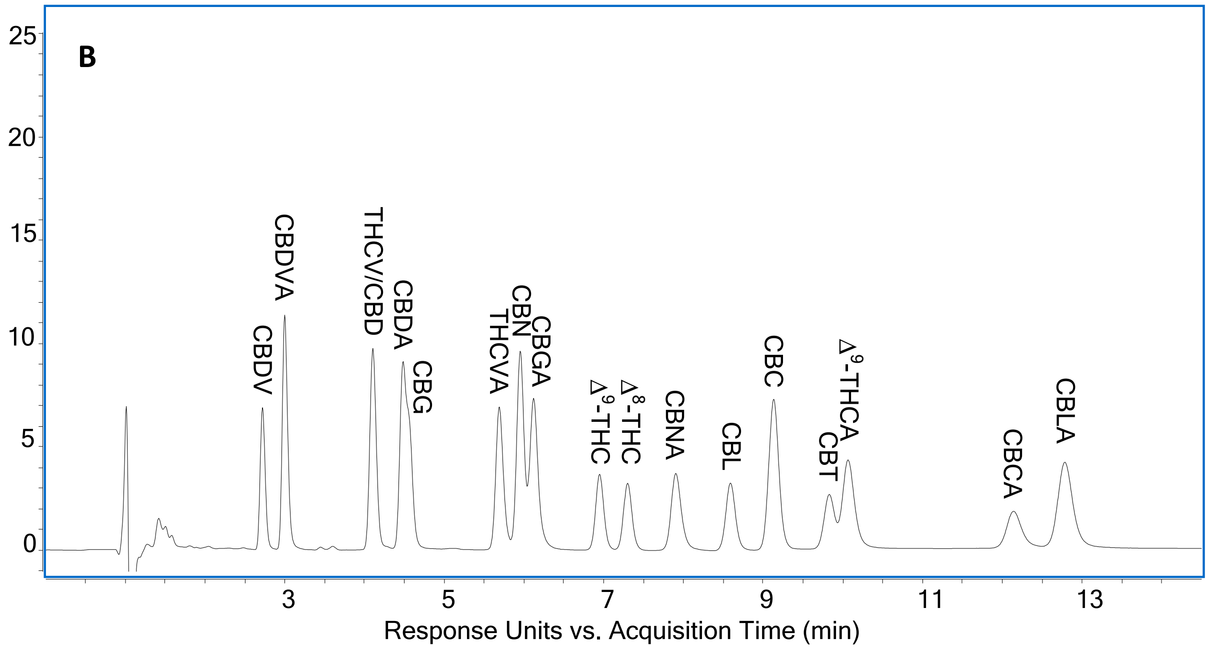
B
25
20
15
10
5
0
CBDV
CBDVA
THCV/CBD
CBDA
CBG
THCVA
CBN
CBGA
Δ9-THC
Δ8-THC
CBNA
CBL
CBC
CBT
Δ9-THCA
CBCA
CBLA
3
5
7
9
11
13
Response Units vs. Acquisition Time (min)

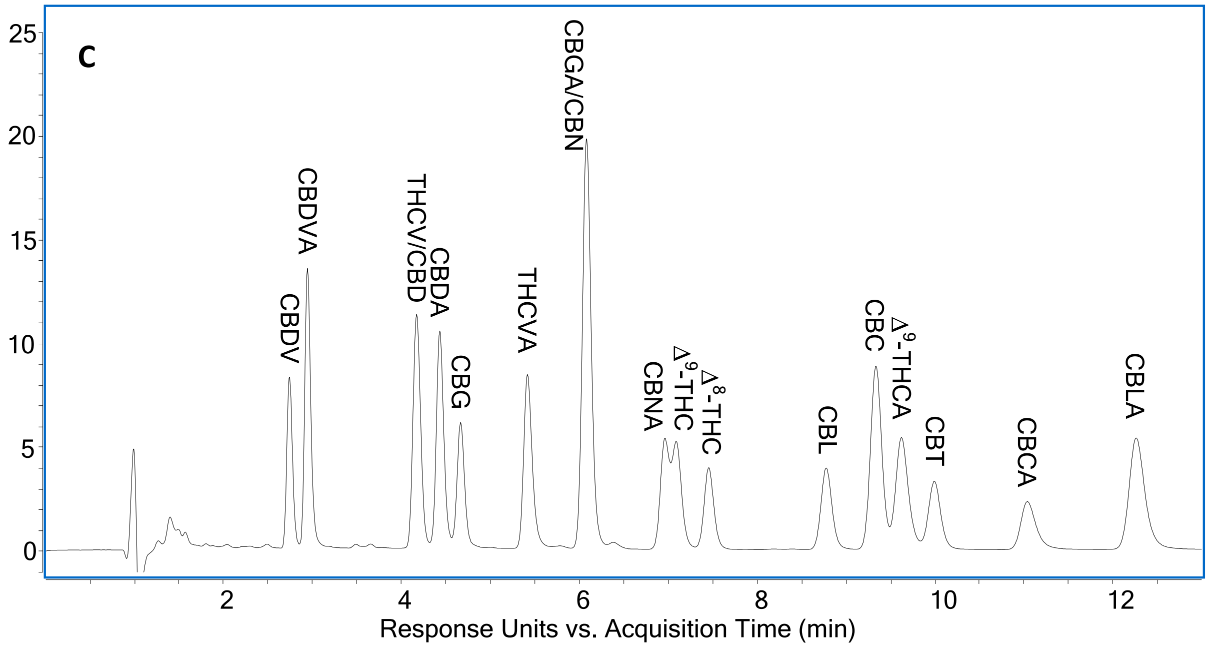
C
25
20
15
10
5
0
CBDV
CBDVA
THCV/CBD
CBDA
CBG
THCVA
CBGA/CBN
CBNA
Δ9-THC
Δ8-THC
CBL
CBC
Δ9-THCA
CBT
CBCA
CBLA
2
4
6
8
10
12
Response Units vs. Acquisition Time (min)

Supplementary Fig. S9. LC separation of eighteen cannabinoids using Cortecs Shield RP-18. The A solvent was 0.01% HCO_2H + 1 mM NH_4HCO_2 (pH 3.38); the B solvent was acetonitrile; the mobile phase contained 70.0% (v/v) B; the flow rate was 0.4 mL/min; the eighteen cannabinoids were at 1 μg/mL individual concentration.

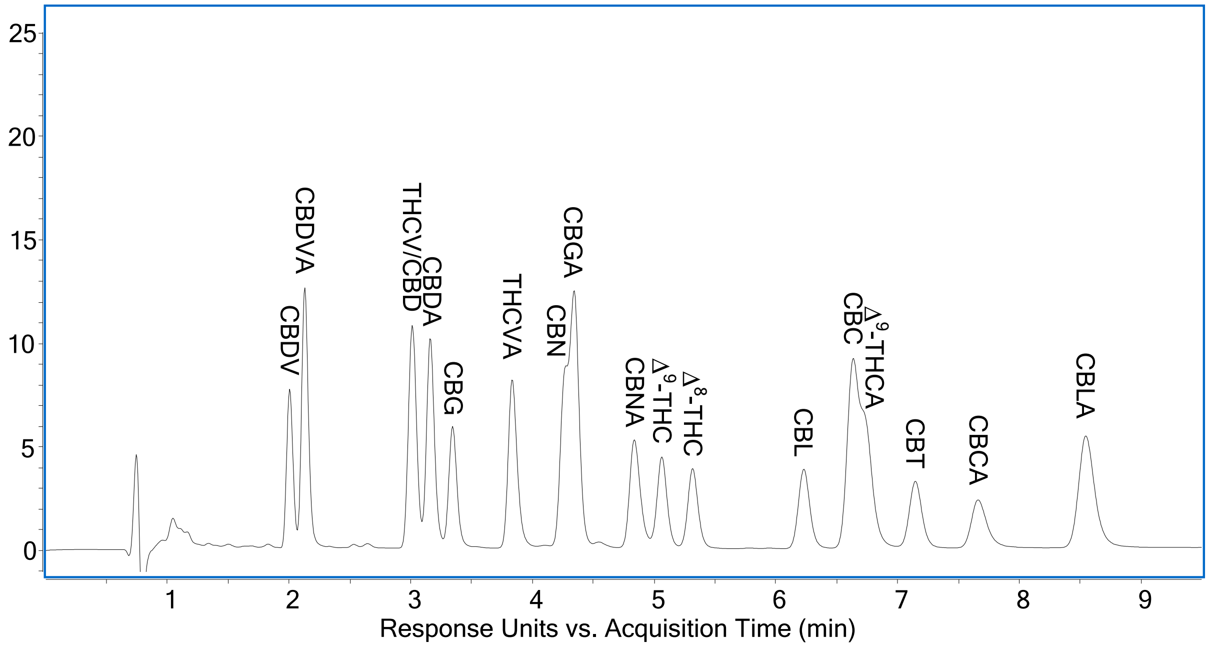

Supplementary Table S1

Published LC-DAD methods that were able to quantify fourteen and more cannabinoids for potency testing of hemp-based products (neutral cannabinoids are in bold font).

Ref.	Column and temperature	Mobile phase	Order of elution
18	Restek Raptor ARC-18<br>150 × 4.6 mm × 2.7 µm	A: 0.1% HCO_2H + 5 mM NH_4HCO_2/H_2O<br>B: 0.1% HCO_2H/MeCN<br>75% B for 11 min	CBDVA, **CBDV**, CBDA, CBGA, **CBG**, **CBD**, **THCV**, THCVA, **CBN**, CBNA, **Δ⁹-THC**, **Δ⁸-THC**, **CBL**, **CBC**, Δ⁹-THCA, CBLA, CBCA
13	Restek Raptor ARC-18<br>2 ×150 × 4.6 mm × 2.7 µm<br>30 ◦C	A: 0.02% HCO_2H + 0.5 mM NH_4HCO_2/H_2O (pH3.0)<br>B: MeCN<br>75% B for 19 min<br>0.4 mL/min	CBDVA, **CBDV**, CBDA, CBGA, **CBG**, **CBD**, **THCV**, **ACBD**, **CBCV**, THCVA, **CBN**, CBNA, **Δ⁹-THC**, **Δ⁸-THC**, **CBL**, **CBC**, Δ⁹-THCA, Δ⁸-THCA, CBCA, CBLA, **CBT**
19	Phenomenex Luna C18(2)<br>250 mm × 4.6 mm × 3 µm<br>40 ◦C.	A: 0.1% HCO_2H/H_2O<br>B: 0.1% HCO_2H/MeCN<br>0-10 min, 75% B, 1 mL/min<br>10.1-30 min, 75% B, 1.2 mL/min	CBDVA, **CBDV**, CBDA, CBGA, **CBG**, **CBD**, **THCV**, THCVA, **CBN**, CBNA, **Δ⁹-THC**, **Δ⁸-THC**, **CBL**, **CBC**, Δ⁹-THCA, CBCA
20	Shimadzu Shim-Pak C18<br>75 mm × 3 mm × 2.2 µm +<br>Phenomenex Synergy C18<br>2 × 100 mm × 3 mm × 2.5 µm<br>40 ◦C	A: 0.1% HCO_2H/MeCN<br>B: 0.1% HCO_2H/H_2O<br>C: MeOH<br>A ternary gradient involving 15 steps to change flow rate, %B and %C	CBDVA, **CBDV**, CBDA, CBGA, **CBG**, **CBD**, **THCV**, THCVA, **CBN**, **Δ⁹-THC**, **Δ⁸-THC**, CBNA, **CBL**, **CBC**, Δ⁹-THCA, CBCA, CBLA
14	Restek Raptor ARC-18<br>150 × 4.6 mm × 2.7 µm<br>30 ◦C	A: 0.015% HCO_2H/H_2O<br>B: 75/25 MeOH/MeCN<br>74.5% B for 17.5 mins, 0.3 mL/min<br>80.5% B for 6.5 min, 0.3 mL/mil	**CBDV**, CBDVA, **CBD**, **CBG**, **THCV**, CBDA, CBGA, **ACBD**, **CBN**, **Δ⁹-THC**, THCVA, Δ⁸-

		74.5% B for 6 mins, 0.5 mL/min<br>74.5% B for 2 mins, 0.3 mL/min	**THC**, **CBL**, **CBC**, CBNA, **CBT**, Δ^9-THCA, CBLA, CBCA

Supplementary Table S2

Published LC-ESI/MS/MS methods that were able to quantify fourteen and more cannabinoids for potency testing of hemp-based products (neutral cannabinoids are in bold font).

Ref.	Column and temperature	Mobile phase	Order of elution
21	ACE C18-Amide<br>100 mm × 2.1 mm × 3 µm<br>40 ◦C	A: 0.1% HCO_2H/H_2O<br>B: 0.1% HCO_2H/MeCN<br>0–5 min, 57-70% B<br>5.0–11.0 min, 70-75% B<br>11.0–13.0 min, 75-80% B<br>13.0–14.0 min, 80-95% B<br>14.0–18.0 min, 98% B<br>18.0–22.0 min, 57% B.<br>0.5 mL/min	**CBDV**, **THCV**, CBDVA, **CBD**, **CBG**, THCVA, **CBN**, **Δ^9-THC**, **Δ^8-THC**, **CBC**, CBDA, **CBL**, CBNA, Δ^9-THCA, CBGA, CBCA, CBLA
22	Supelco Ascentis Express C18<br>150 mm × 3.0 mm × 2.7 µm<br>25 ◦C	A: 0.1% HCO_2H + 2 mM NH_4HCO_2/H_2O<br>B: 0.1% HCO_2H/MeCN<br>0-20 min, 70-90% B<br>0.3 mL/min	CBGV, **CBDV**, CBDA, CBGA, **CBG**, **CBNR**, **CBD**, **CBCV**, **CBN**, CBCA, **Δ^9-THC**, **Δ^8-THC**, **CBC**, Δ^9-THCA
23	Waters Acquity UPLC BEH C18<br>150 mm × 2.1 mm × 1.7 µm<br>30 ◦C	A: 0.1% HCO_2H/H_2O<br>B: 0.1% HCO_2H/MeCN<br>0-1.5 min, 40–70% B<br>1.5-15.5 min, 70–100% B<br>15.5-16 min, 100–40% B<br>16.0-18.0 min, 40% B<br>0.3 mL/min	CBDVA, **CBDV**, CBDA, **CBD**, **CBG**/CBGA, **THCV**, **CBN**/THCVA, **Δ^9-THC**, CBNA, **CBL**, **CBC**, Δ^9-THCA, CBCA, CBLA
24	Phenomenex Luna Omega C18<br>150 mm × 2.1 mm × 1.6 µm<br>40 ◦C	A: 0.1% HCO_2H/H_2O<br>B: 0.1% HCO_2H/MeCN<br>0-5 min, 70% B<br>5–7 min, 100% B<br>7–7.1 min, 70% B	CBDVA, **CBDV**, CBDA, CBGA, **CBG**, **CBD**, **THCV**, THCVA, **CBN**, CBNA, **Δ^9-THC**/**Δ^8-THC**, **CBL/CBC**, Δ^9-THCA, CBCA

		7.1–8 min, 70% B<br>0.4 mL/min	
15	Phenomenex Luna Omega Polar C18<br>150 mm × 2.1 mm × 1.6 µm<br>30 ◦C	A: 0.011% HCO_2H + 2 mM NH_4HCO_2/H_2O (pH3.6)<br>B: MeCN<br>73% B for 18 min<br>0.3 mL/min	CBDVA, **CBDV**, CBDA, CBGA, CBNA, THCVA, **CBG**, **CBD**, **THCV**, **ACBD**, **CBN**, CBCA, Δ^9-THCA, **Δ^9-THC**, **Δ^8-THC**, CBLA, **CBL**, **CBC**, **CBT**

Supplementary Table S3

Published LC-ESI/MS methods that were able to quantify fourteen and more cannabinoids for potency testing of hemp-based products (neutral cannabinoids are in bold font).

Ref.	Column and temperature	Mobile phase	Order of elution
25	Waters Cortecs C18<br>2.1 mm × 100 mm × 1.6 µm<br>20 ◦C	A: 0.02% HCO_2H + 5 mM NH_4HCO_2/H_2O<br>B: MeCN<br>75% B for 10 min<br>0.3 mL/min.	CBDVA, **CBDV**, CBDA, CBGA, **CBG**, **CBD**, CBNA, THCVA, **THCV**, **CBN**, CBCA, **Δ^9-THC**, Δ^9-THCA, **Δ^8-THC**, CBLA
26	Agilent Poroshell C18<br>50 mm × 2.1 mm × 2.7 µm<br>30 ◦C	A: 0.1% HCO_2H/30%MeOH/H_2O<br>B: 0.1% HCO_2H/MeCN<br>0–25 min, 53% B<br>5–40 min, 53–70% B<br>40–42 min, 70–97% B<br>42–43 min, 97%B<br>43–44 min, 97-53%B<br>44–50 min, 53%B<br>0.3 mL/min	**CBDV**, CBDVA, CBDA, **CBD/CBG**, **THCV**, CBGA, THCVA, **CBN**, **Δ^9-THC**, **Δ^8-THC**, **CBL**, CBNA, **CBC**, Δ^9-THCA, CBLA, CBCA

Supplementary Table S4

Theoretical plate numbers (N) of the ten neutral cannabinoids using 70.0% (v/v) acetonitrile in the mobile phase. The A solvent was 0.02% (v/v) HCO_2H.

Column	Cortecs	Raptor	Poroshell	Ascentis
CBDV	8521	10879	14085	11907
CBG	18789	23022	24724	25349
CBD	15145	18988	23390	19440
THCV	15224	19677	24224	17919
CBN	20950	28826	32273	27352
Δ9-THC	24566	30586	32566	27262
Δ8-THC	28177	38576	37029	28438
CBL	33434	41562	38260	37541
CBC	33784	45645	38570	38218
CBT	27413	37783	35289	23044
